# A metabolomics perspective on clorobiocin biosynthesis: discovery of bromobiocin and novel derivatives through LC-MS^E^-based molecular networking

**DOI:** 10.1128/spectrum.00423-24

**Published:** 2024-06-12

**Authors:** Niklas B. M. Janzing, Maurice Niehoff, Wolfram Sander, Christoph H. R. Senges, Sina Schäkermann, Julia E. Bandow

**Affiliations:** 1 Applied Microbiology, Faculty of Biology and Biotechnology, Ruhr University Bochum, Bochum, Germany; 2 Organic Chemistry II, Faculty of Chemistry and Biochemistry, Ruhr University Bochum, Bochum, Germany; University of Melbourne, Melbourne, Australia

**Keywords:** metabolomics, specialized metabolite production, metabolic network, halogenase, antibiotic

## Abstract

Clorobiocin is a well-known, highly effective inhibitor of DNA gyrase belonging to the aminocoumarin antibiotics. To identify potentially novel derivatives of this natural product, we conducted an untargeted investigation of clorobiocin biosynthesis in the known producer *Streptomyces roseochromogenes* DS 12.976 using LC-MS^E^, molecular networking, and analysis of fragmentation spectra. Previously undescribed clorobiocin derivatives uncovered in this study include bromobiocin, a variant halogenated with bromine instead of chlorine, hydroxylated clorobiocin, carrying an additional hydroxyl group on its 5-methyl-pyrrole 2-carboxyl moiety, and two other derivatives with modifications on their 3-dimethylallyl 4-hydroxybenzoate moieties. Furthermore, we identified several compounds not previously considered clorobiocin pathway products, which provide new insights into the clorobiocin biosynthetic pathway. By supplementing the medium with different concentrations of potassium bromide, we confirmed that the clorobiocin halogenase can utilize bromine instead of chlorine. The reaction, however, is impeded such that non-halogenated clorobiocin derivatives accumulate. Preliminary assays indicate that the antibacterial activity of bromobioin against *Bacillus subtilis* and efflux-impaired *Escherichia coli* matches that of clorobiocin. Our findings emphasize that yet unexplored compounds can be discovered from established strains and biosynthetic gene clusters by means of metabolomics analysis and highlight the utility of LC-MS^E^-based methods to contribute to unraveling natural product biosynthetic pathways.

**IMPORTANCE:**

The aminocoumarin clorobiocin is a well-known gyrase inhibitor produced by the gram-positive bacterium *Streptomyces roseochromogenes* DS 12.976. To gain a deeper understanding of the biosynthetic pathway of this complex composite of three chemically distinct entities and the product spectrum, we chose a metabolite-centric approach. Employing high-resolution LC-MS^E^ analysis, we investigated the pathway products in extracted culture supernatants of the natural producer. Novel pathway products were identified that expand our understanding of three aspects of the biosynthetic pathway, namely the modification of the noviose, transfer and methylation of the pyrrole 2-carboxyl moiety, and halogenation. For the first time, brominated products were detected. Their levels and the levels of non-halogenated products increased in medium supplemented with KBr. Based on the presented data, we propose that the enzyme promiscuity contributes to a broad product spectrum.

## INTRODUCTION

The emergence of antimicrobial resistances necessitates urgent efforts to develop novel antibiotics. Natural products have proven to be an invaluable source of bioactive molecules, with over 70% of antibacterial drugs developed in the past four decades being natural products or derivatives thereof (1). Actinobacteria, and in particular the genus *Streptomyces*, are responsible for the production of a wide array of antibiotic classes (2). In the recent past, our knowledge about Actinobacteria grew profoundly. Genomic analyses revealed that the biosynthetic potential of many strains exceeds expectations founded in activity or liquid chromatography-based compound discovery efforts, and it is estimated that up to 90% of the genetic potential remains undiscovered (3). Much of this untapped potential resides in silent or cryptic biosynthetic gene clusters which require specialized cultivation techniques or genetic manipulation for activation (4). Global metabolomic approaches enable comparative analyses of a strain’s metabolome under different cultivation conditions, facilitating the deduction of cultivation parameters conducive to the production of specific compounds (5). Moreover, modern mass spectrometry techniques offer insights into the chemical structure of molecules and contribute to elucidating biosynthetic pathways (6). This accelerates natural product research, e.g., by allowing to focus purification efforts, which continue to present a common bottleneck.

**Fig 1:**
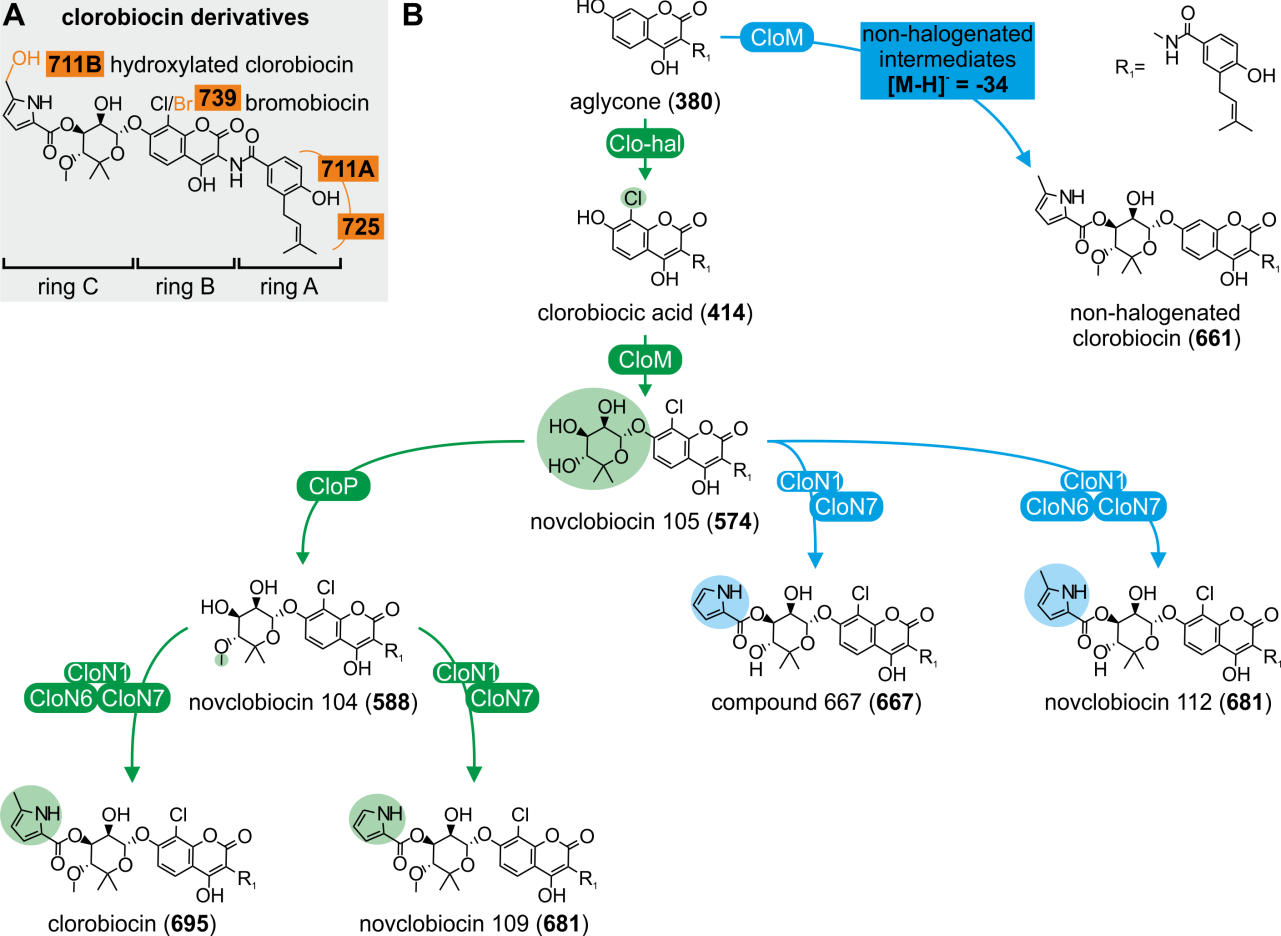
Clorobiocin and structurally related molecules detected in the supernatant of *S. roseochromogenes* DS 12.976. The [M-H]^−^ of each molecule is given in brackets. (**A**) Previously undescribed clorobiocin derivatives proposed based on this study. The clorobiocin derivatives were structurally annotated using fragmentation spectra analysis (711A, 711B, 725, 739) and nuclear magnetic resonance spectroscopy (711B). Modifications deviating from the clorobiocin molecule are highlighted in orange. (**B**) Proposed biosynthetic pathway of clorobiocin following its aglycone formation. Structural modifications that are added by the enzymatic steps are indicated in green circles. The proposed alternative enzymatic routes leading to the formation of different shunt products are indicated in blue arrows. Structural modifications added are indicated in blue circles. Data in (**B**) in part from Melnyk *et al*. (20) and Kammerer *et al*. (21).

Clorobiocin is an aminocoumarin antibiotic produced and secreted by *Streptomyces roseochromogenes* DS 12.976. It selectively inhibits the bacterial DNA gyrase and topoisomerase IV by targeting their ATPase subunits (GyrB) and thereby impeding DNA supercoiling, replication, and transcription. With an equilibrium dissociation constant (*K_D_*) below 5 nM, the target-binding efficacy of clorobiocin (7, 8) by far exceeds that of the clinically used fluoroquinolones which target DNA gyrase GyrA subunits (9) with *K_D_*s in the micromolar range (10). Since the initial identification of the clorobiocin biosynthetic gene cluster (11), significant progress has been made elucidating the biosynthetic pathway. As of today, nearly all genes involved have been fully characterized. Furthermore, numerous structure-activity relationship studies explored the antibacterial potential of clorobiocin and derivatives generated using biochemical, genetic, and synthetic approaches (12–18). Clorobiocin is composed of a tripartite structure: a prenylated 4-hydroxybenzoyl moiety (ring A), a central 3-amino 4,7-dihydroxycoumarin moiety (ring B), and a modified deoxy sugar known as noviose (ring C) (Fig. 1A). The synthesis and assembly of these rings are facilitated through a series of enzymatic reactions. Ring A is derived from prephenate, which is modified by CloFQR. Ring B originates from _L_-tyrosine that undergoes transformation by CloHUJK. Ring C is sourced from glucose 1-phosphate that is enzymatically modified by CloVTWUS. Rings A and B are linked by the amide synthetase CloL. Following this, Clo-hal chlorinates the C-8′ position of the aminocoumarin, yielding clorobiocic acid. Subsequently, ring C is attached to clorobiocic acid by the glycosyltransferase CloM, resulting in the formation of novclobiocin 105. After the assembly of the clorobiocin core components, further modifications take place. CloP methylates the 4′-OH position of ring C, while CloN4, CloN5, and CloN3 collectively orchestrate the synthesis of the pyrrole 2-carboxyl moiety from _L_-proline. The final methylation and transfer of this moiety to the 3′-OH position of ring C are then carried out by CloN1, CloN6, and CloN7 [see the study by Heide (19) for a comprehensive review].

In this work, building on a previous LC-MS-based study, which identified intermediates and derivatives of the clorobiocin pathway (21), we investigated the production of clorobiocin in *S. roseochromogenes* DS 12.976. Here, instead of a bottom-up approach specifically screening for predicted clorobiocin-related intermediates, we utilized a top-down strategy enabling us to obtain a global view of the metabolome and explore uncharted aspects of clorobiocin biosynthesis. In this endeavor, we analyzed extracts of *S. roseochromogenes* DS 12.976 culture supernatants using LC-MS^E^ technology and then used the Global Natural Products Social Molecular Networking (GNPS) ecosystem (22) to generate a molecular network based on the similarity of fragmentation spectra. Analyzing the clorobiocin subnetwork, we identified all previously known clorobiocin pathway intermediates that follow the aglycone formation, as well as additional clorobiocin-related compounds (Fig. 1) and novel brominated and hydroxylated clorobiocin derivatives (Fig. 1A).

## MATERIALS AND METHODS

### Strains and cultivation conditions

*Streptomyces roseochromogenes* DS 12.976 was routinely cultivated in 100 mL Erlenmeyer flasks containing 10 mL DSMZ 65 (https://mediadive.dsmz.de) complex medium (4 g/L glucose, 4 g/L yeast extract, 10 g/L malt extract) at 30°C under constant agitation (180 rpm). To investigate the influence of different halogens on clorobiocin production, the medium was supplemented with KBr (0.025%, 0.05%, 0.1%, and 0.2%), sodium fluoride (NaF) (0.1%), or potassium iodide (KI) (0.1%) prior to cultivation (w/v). To generate molecular networks, to isolate clorobiocin and clorobiocin derivatives, and for time-resolved analyses, *S. roseochromogenes* DS 12.976 was precultured for 3 days in 100 mL Erlenmeyer flasks containing 10 mL DSMZ 65 medium, prior to inoculating 3 L Fernbach flasks with 1 L of DSMZ 65 medium with two precultures each. Main cultures were incubated for 7 days at 30°C under constant agitation (180 rpm). For the production of bromobiocin, non-halogenated clorobiocin, and derivatives, the final cultivation medium was supplemented with 0.2% KBr (w/v) prior to cultivation. For time-resolved analyses, samples of 1 mL were taken daily from main cultures and stored at −20°C until further use. All experiments were performed in biological triplicates.

### Compound extraction

For MS analysis, 500 µL of culture supernatant were harvested and extracted with 1 mL of ethyl acetate (EtOAc). The organic phase was separated, washed twice with 200 µL water, and dried *in vacuo*. All residues were reconstituted in 100 µL methanol (MeOH) and used for MS analysis.

For the generation of molecular networks and preparative purposes, 6 L fermentation broth were extracted with equal volumes of EtOAc. The organic phase was evaporated, and the crude extract was loaded onto silica gel and further separated with a CombiFlash EZ Prep system equipped with a PurIon mass spectrometer (Teledyne Technologies, Thousand Oaks, CA, USA) and a 40-g silica column (RediSepRf, Teledyne Technologies). Using a dichloromethane (CH_2_Cl_2_)/MeOH gradient with a flow rate of 30 mL/min (linear gradient from 2% to 98% MeOH over 10 min) for separation, clorobiocin-like compounds were detected at 338 nm triggering fraction collection. The collected fractions were pooled, evaporated, and reconstituted in 20 mL MeOH. For the generation of molecular networks, 1:10 dilutions were prepared, and 5 µL were injected for LC-MS^E^ measurements. For preparative purposes, the reconstituted isolated fractions were further purified. To isolate clorobiocin, its precursors, and derivatives, a 1260 Infinity II Preparative LC/MSD system (Agilent Technologies, Ratingen, Germany) was utilized. An InfinityLab Poroshell 120 SB-C18 column (particle size 4 µm, column dimensions: 21.2 × 150 mm, Agilent Technologies) with a water/acetonitrile (ACN) gradient with 0.1% formic acid (FA) was used with a flow rate of 30 mL/min (isocratic elution at 59% ACN for 1 min, linear gradient from 59% to 75% ACN over 6 min, and isocratic elution at 100% ACN for 3 min). MS-guided compound detection and fractionation were done in negative ionization mode. All reagents used were HPLC grade. From the fermentation broth of *S. roseochromogenes* DS 12.976, 4 mg/L of clorobiocin, 0.3 mg/L of derivative 711B, and 0.35 mg/L of novclobiocin 104 were isolated. When utilizing KBr-supplemented cultivation broth, yields were as follows: 3.1 mg/L for non-halogenated clorobiocin, 0.25 mg/L for a mixture of clorobiocin and bromobiocin, and 1 mg/L for non-halogenated derivative 711B (H-677).

### LC-MS^E^ measurements

Samples of 5 µL were injected into an ACQUITY UPLC I-Class System (Waters, Milford, MA, USA) equipped with an ACQUITY UPLC HSST3 column (particle size 1.8 µm, column dimensions: 2.1 × 100 mm, Waters). A water/ACN gradient with 0.1% FA was used with a flow rate of 0.6 mL/min (isocratic elution at 2% ACN for 2 min, linear gradient from 2% to 99.5% ACN over 7 min, isocratic elution at 99.5% ACN for 2 min, linear gradient from 99.5% to 2% ACN over 2 min, and isocratic elution at 2% ACN for 1 min). Data-independent MS^E^ measurements were performed with a Vion IMS QToF mass spectrometer (Waters) with an ESI source in negative sensitivity mode. Masses in a range of 50–2,000 m/z were detected with 0.2 s per scan, and leucine enkephalin was injected as a reference mass every 5 min. Used parameters were capillary voltage 0.8 kV, sample cone voltage 40 V, source offset voltage 80 V, cone gas flow 50 L/h, desolvation gas flow 1,000 L/h, source temperature 150°C, desolvation temperature 550°C, collision gas N_2_, collision low energy 6 V, collision high energy ramp 28–60 V.

Peak picking and lock mass correction were performed using the UNIFI software (version 1.9.13.9, Waters) with the following parameters: automatic chromatographic peak width, automatic peak detection threshold, low energy intensity threshold 200 counts, high energy intensity threshold 700 counts, chromatographic peak width to apply during cluster creation 0.5, chromatographic peak width to apply during high to low energy association 0.5, drift peak width to apply during cluster creation 0.5, drift peak width to apply during high to low energy association 0.5, intensity threshold to apply during high to low energy association 50, maximum considered charge 1, maximum number of isotopes 3, minimum allowed monoisotopic to largest isotope ratio 0.7, allow wider tolerance for saturated data.

### Computation of mass spectral networks and spectra annotation

Files were converted from .mgf to .mzXML using Proteowizard (version 3.0.9490) (23), with 32-bit binary encoding precision and uploaded to the Global Natural Products Social Molecular Networking platform (gnps.ucsd.edu) (22). For the creation of a molecular network based on fragmentation spectra, the METABOLOMICS_SNETS 18 workflow was used with the following parameters: parent mass tolerance 0.02 Da, ion tolerance 0.05 Da, minimal pairs cos 0.7, network topK 10, maximum connected component size 100, minimum cluster size 2, run MSCluster. The generated network was visualized in Cytoscape (version 3.6.0) (24) and processed by manual dereplication. Redundancies were cleared manually. Compounds within the molecular subnetwork of clorobiocin were identified and annotated by their molecular masses and fragmentation spectra. Fragmentation spectra were annotated and aided by the MetFrag *in silico* fragmentation tool (25).

### Structure elucidation by nuclear magnetic resonance spectroscopy

The nuclear magnetic resonance (NMR) spectra of the isolated clorobiocin derivative 711B (approx. 3.5 µmol/mL) were recorded using an AV III 400 (400 MHz) instrument (Bruker Daltonik, Billerica, MA, USA). MeOH-d_4_ (Eurisotop, Saint-Aubin, France) was used as solvent. The spectra obtained were referenced using the residual signal CD_3_OD: δ (1H) = 4.57 ppm. MestreNova 14.2.1 purchased from Mestrelab Research S.L. (Santiago de Compostela, Spain) was used to process the spectra.

### MIC determination and disk diffusion assays

Minimal inhibitory concentrations (MIC) against *B. subtilis* 168 and *E. coli* EP1581 were determined in microtiter plates. The test organisms were grown in 5 mL Mueller-Hinton bouillon (Carl Roth, Karlsruhe, Germany) at 37°C under constant agitation (200 rpm). At an OD_600_ of 1.0 [*B. subtilis*: 6 × 10^7^ colony-forming units (cfu)/mL; *E. coli*: 2 × 10^9^ cfu/mL]), the cultures were used to prepare microtiter wells with 5 × 10^5^ cfu/mL in 198 µL Mueller-Hinton bouillon. Compounds of interest dissolved in MeOH were prepared such that addition of 2 µL yielded desired final concentrations to be tested in the 200 µL assay volume. Microtiter plates were incubated overnight at 37°C. The MIC was defined as the lowest concentration that inhibited visible bacterial growth.

For the disk diffusion assay, test organisms were grown to an OD_600_ of 1.0 as described above. Furthermore, 50 mL of still liquid LB agar (yeast extract 5 g/L, tryptone 10 g/L, NaCl 5 g/L, agar 20 g/L) were inoculated with 1.25 mL of test culture and poured into square plastic dishes for solidification. Filter paper disks with 10 µg of each test compound were placed onto the agar. Plates were incubated overnight at 37°C.

## RESULTS

### Molecular networking reveals the clorobiocin spectral family

**Fig 2:**
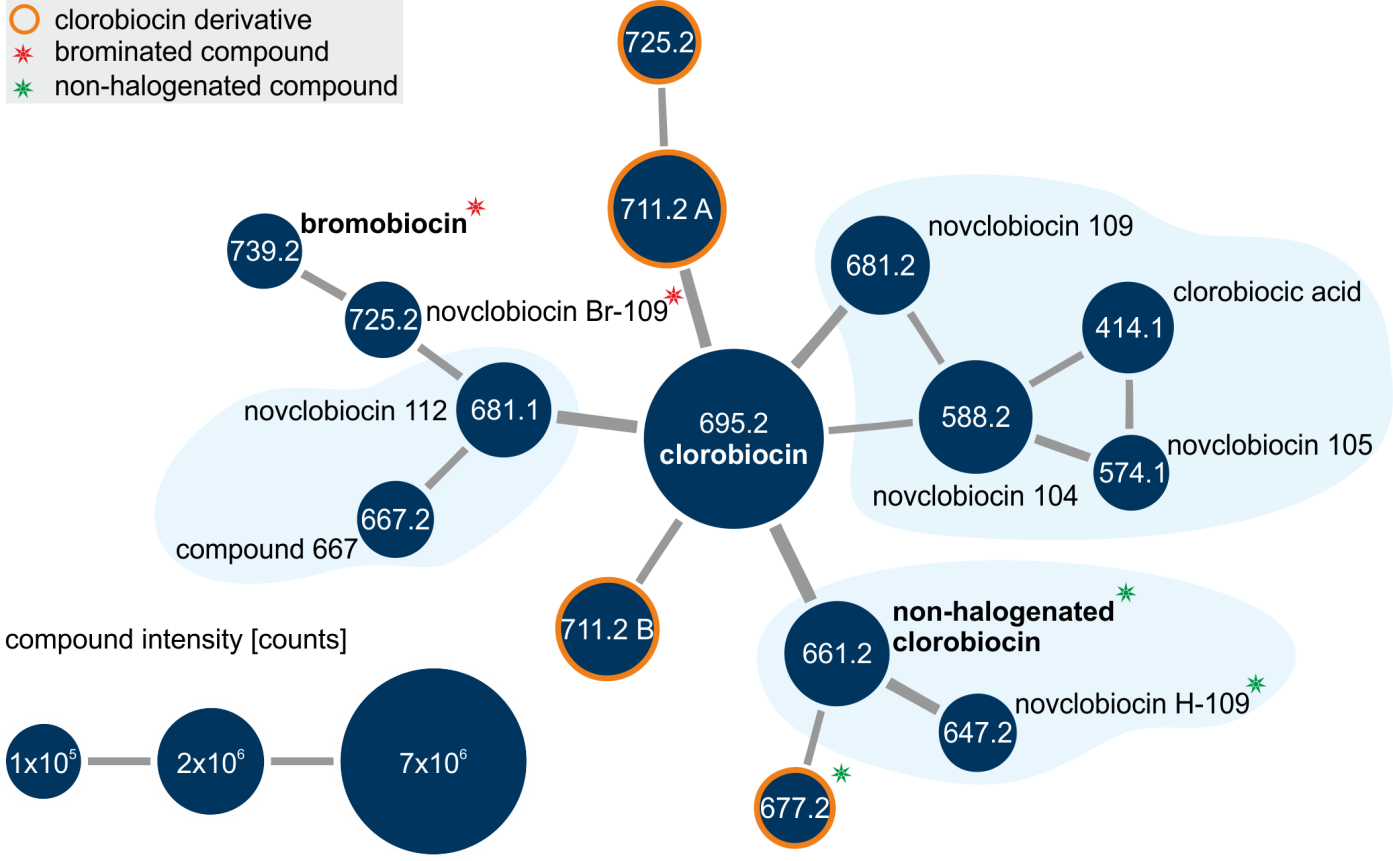
Spectral family of clorobiocin. Extracts of culture supernatant of *S. roseochromogenes* DS 12.976 grown in DSMZ 65 complex medium were analyzed by LC-MS^E^. From the generated molecular network, the clorobiocin subnetwork was studied in detail. Nodes represent metabolites with their respective molecular masses ([M-H]^−^), and node sizes indicate the accumulated abundance. Compounds are connected when the similarity score was at least 0.7. The identity of the clorobiocin-related compounds was assigned based on structural features delineated from their fragmentation spectra. Compounds with molecular masses greater than that of clorobiocin or with novel structural features were classified as clorobiocin derivatives. Compounds containing a bromine instead of a chlorine atom are marked with a red asterisk, those lacking halogenation with a green asterisk. “Br-” or “H-” precedes the numbers in their name, respectively.

*S. roseochromogenes* DS 12.976 was cultivated on a scale of several liters in DSMZ 65 medium for 7 days. The organic extract obtained from the supernatant was subjected to flash chromatography. Fractions containing clorobiocin-like compounds were evaporated, reconstituted in MeOH, and subjected to LC-MS^E^ analysis. Subsequently, the GNPS molecular networking workflow (22) was used to generate a molecular network based on the similarity of fragmentation spectra. Within this molecular network, clorobiocin and structurally related molecules formed a subnetwork that was further investigated to annotate known and yet uncharacterized compounds (Fig. 2).

**Fig 3:**
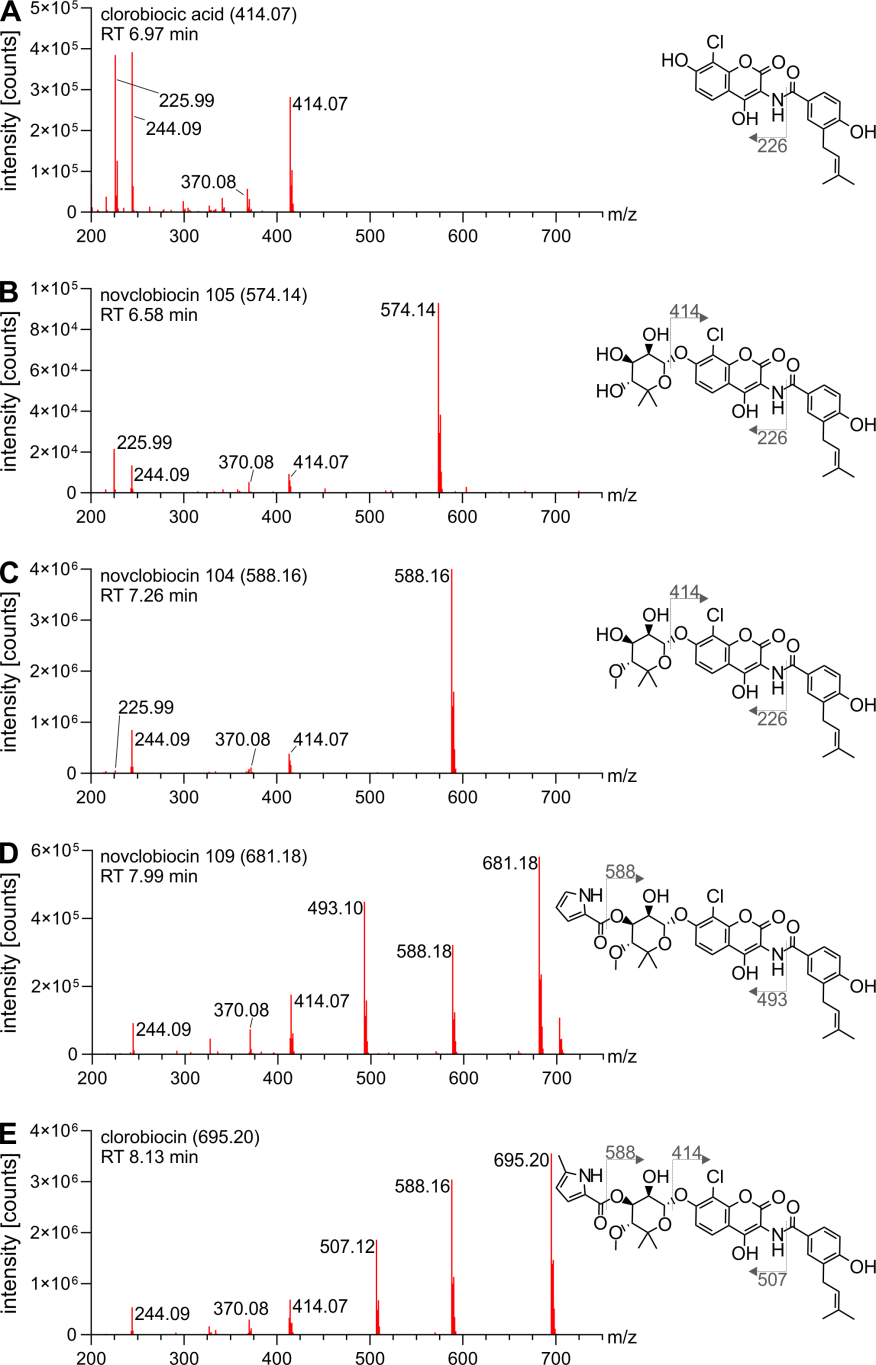
Fragmentation spectra of clorobiocin pathway products. Clorobiocin and clorobiocin-like compounds were extracted from the culture supernatant of *S. roseochromogenes* DS 12.976 and analyzed using LC-MS^E^. Fragment peaks of detected compounds were assigned to proposed structures using the clorobiocin fragmentation spectrum (E) as reference. The [M-H]^−^ of the parental masses for clorobiocic acid (A), novclobiocin 105 (B), novclobiocin 104 (C), novclobiocin 109 (D), and clorobiocin (E) are given in brackets following the trivial name. Fragmentation sites are denoted by dotted lines, and [M-H]^−^ of fragments are indicated.

As expected, clorobiocin (695.2 [M-H]^−^) was the most abundant compound within its spectral family. Apart from clorobiocin, a total of 14 other compounds were detected. To annotate the compounds in the clorobiocin subnetwork, we compared the mono-isotopic masses of known clorobiocin pathway intermediates with the experimentally observed masses (Fig. 3). Moreover, we analyzed the fragmentation spectra of the compounds using that of clorobiocin as a reference (see Table S1 for a summary of the MS-based annotations and chemical cross sections).

With our LC-MS^E^ settings, the fragmentation spectrum of clorobiocin featured three major fragment peaks (Fig. 3E), each corresponding to a distinct part of the clorobiocin molecule. The most prominent fragment peak at 588.16 [M-H]^−^ was a result of the loss of the 5-methyl-pyrrole 2-carboxyl moiety of ring C, whereas the fragment peaks at 414.07 and 507.11 [M-H]^−^ were caused by the loss of the entire ring C or ring A, respectively (Fig. 1A shows rings A–C of the clorobiocin molecule). Moreover, the presence of a chlorine atom within a molecule or fragment was confirmed by the distinct isotopic pattern resulting from the chlorine isotopes ^35^Cl and ^37^Cl. Since these have a natural abundance ratio of approximately 3:1, chlorinated molecules display two characteristic molecular ion peaks that have a similar intensity ratio of 3:1 and differ by two atomic mass units.

### Identification of clorobiocin pathway intermediates and shunt products

The first set of identified pathway intermediates consists of compounds 414.1, 574.1, 588.2, and 681.2 [M-H]^−^ (Fig. 2). They were connected by edges, indicating their high structural similarity, and their mass spectra had characteristic chlorine isotope patterns. Based on its total mass and fragment masses, compound 414.1 [M-H]^−^ was identified as clorobiocic acid, the halogenated clorobiocin aglycone (Fig. 3A). Similarly, compound 574.1 [M-H]^−^, displaying the same fragment peaks as clorobiocic acid, was identified as novclobiocin 105 [M-H]^−^ (Fig. 3B). Novclobiocin 105 [M-H]^−^ results from the glycosylation of clorobiocic acid with noviose, and subsequent methylation at the 4′-OH position transforms it into novclobiocin 104, identified as compound 588.2 [M-H]^−^ (Fig. 3C). Novclobiocin 104 and 105 also shared most of the characteristic fragment peaks of clorobiocic acid (225.99, 244.90, 370.08, and 414.07). Finally, compound 681.2 [M-H]^−^ exhibited a fragmentation spectrum and molecular mass consistent with novclobiocin 109. This compound is acylated with the pyrrole 2-carboxyl moiety at the 3′-OH position of the noviose sugar but lacks methylation of this moiety (Fig. 3D).

**Fig 4:**
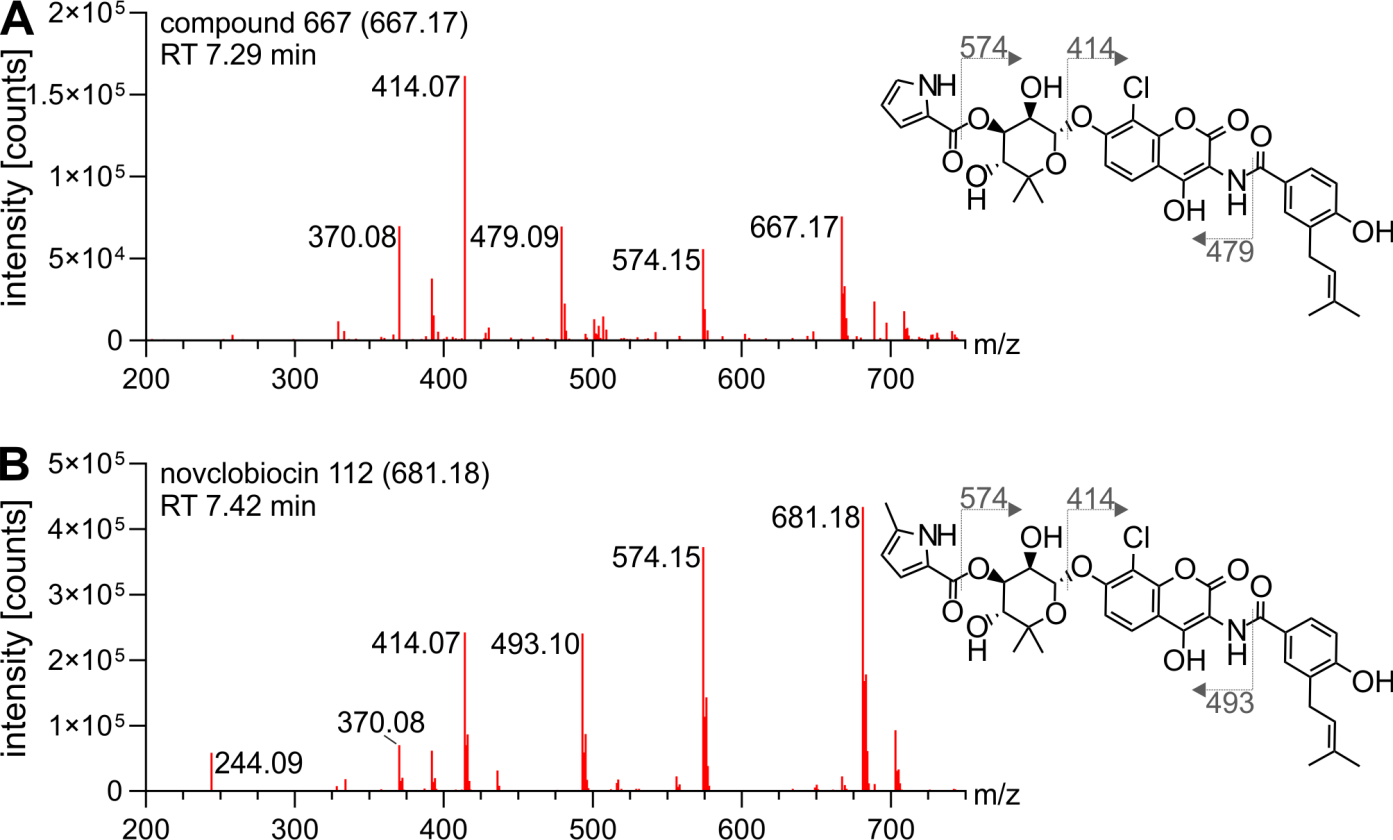
Fragmentation spectra of compound 667.2 [M-H]^−^ (A) and novclobiocin 112 (B). Clorobiocin and clorobiocin-like compounds were extracted from the culture supernatant of *S. roseochromogenes* DS 12.976 and analyzed using LC-MS^E^. Fragment peaks of detected compounds were assigned to proposed structures using the clorobiocin fragmentation spectrum (Fig. 3E) as a reference. The [M-H]^−^ of parental masses are given in brackets following the trivial name. Fragmentation sites are denoted by dotted lines, and [M-H]^−^ of fragments are indicated.

Compounds 681.1 and 667.2 [M-H]^−^ constitute the second group of clorobiocin pathway products (Fig. 2). Compound 667.2 [M-H]^−^ was identified as a clorobiocin-related compound having an unmethylated noviose moiety as well as an unmethylated pyrrole 2-carboxyl moiety (Fig. 4A). This was evidenced by the presence of the 414.07 [M-H]^−^ fragment confirming unaltered rings A and B. On the other hand, the 574.14 and 507.11 [M-H]^−^ fragments showed that neither ring C nor the pyrrole 2-carboxyl moiety was methylated. Similarly, compound 681.1 [M-H]^−^ was identified as novclobiocin 112 which, in comparison to clorobiocin, only lacks the 4′-OH methylation of its noviose moiety (Fig. 4B). This was supported by an unchanged 414.07 [M-H]^−^ fragment, matching unaltered rings A and B, and a 574.14 [M-H]^−^ fragment, showing the absence of a methylation on the noviose, which would have resulted in a 588.16 [M-H]^−^ fragment (as seen with clorobiocin, Fig. 3E).

**Fig 5:**
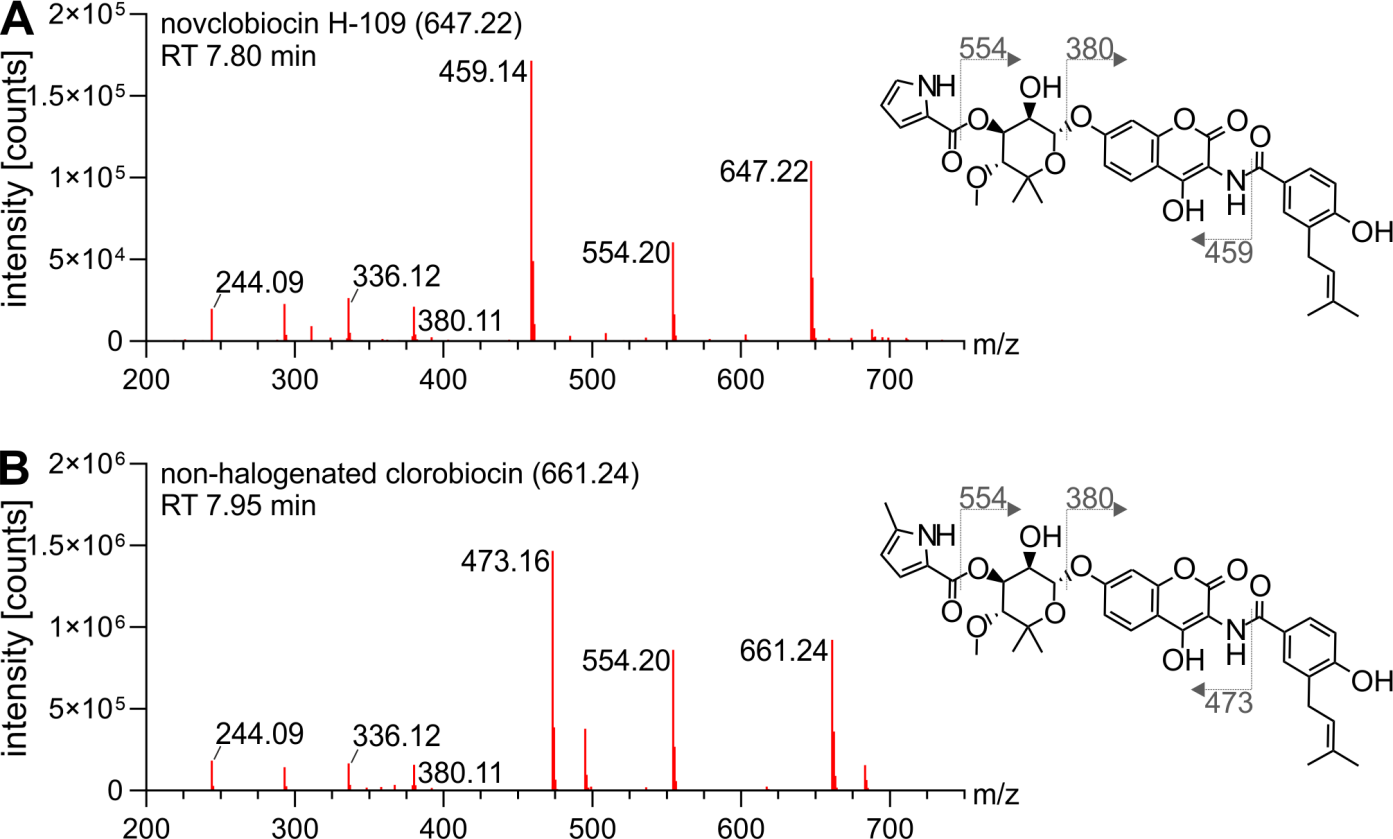
Fragmentation spectra of non-halogenated novclobiocin H-109 (A) and non-halogenated clorobiocin (B). Clorobiocin and clorobiocin-like compounds were extracted from the culture supernatant of *S. roseochromogenes* DS 12.976 and analyzed using LC-MS^E^. Fragment peaks of detected compounds were assigned to proposed structures using the clorobiocin fragmentation spectrum as a reference. The [M-H]^−^ of parental masses are given in brackets following the trivial names. Fragmentation sites are denoted by dotted lines, and [M-H]^−^ of fragments are indicated.

Compounds 647.2 and 661.2 [M-H]^−^ (Fig. 2) lacked the previously seen distinct isotope patterns characteristic for chlorinated molecules and were therefore identified as non-chlorinated shunt products. Compound 647.2 [M-H]^−^ was identified to be a non-halogenated variant of novclobiocin 109 (novclobiocin H-109). This was evidenced by the previously seen characteristic fragmentation spectrum (Fig. 3D), which was now shifted in correspondence to the absence of a chlorine atom (Fig. 5A). The same was true for compound 661.1 [M-H]^−^, which was identified as non-halogenated clorobiocin, previously described as novclobiocin 101 [13] (Fig. 5B).

### Identification of novel clorobiocin derivatives 711A, 711B, and 725 [M-H]^−^

Molecules within the clorobiocin spectral family (Fig. 2) that had a higher molecular mass than clorobiocin or novel structural features were classified as clorobiocin derivatives. Those included two compounds with the [M-H]^−^ of 711.19 (A + B) and one compound with the [M-H]^−^ of 725.21. All of them were identified as clorobiocin derivatives carrying different structural modifications.

**Fig 6:**
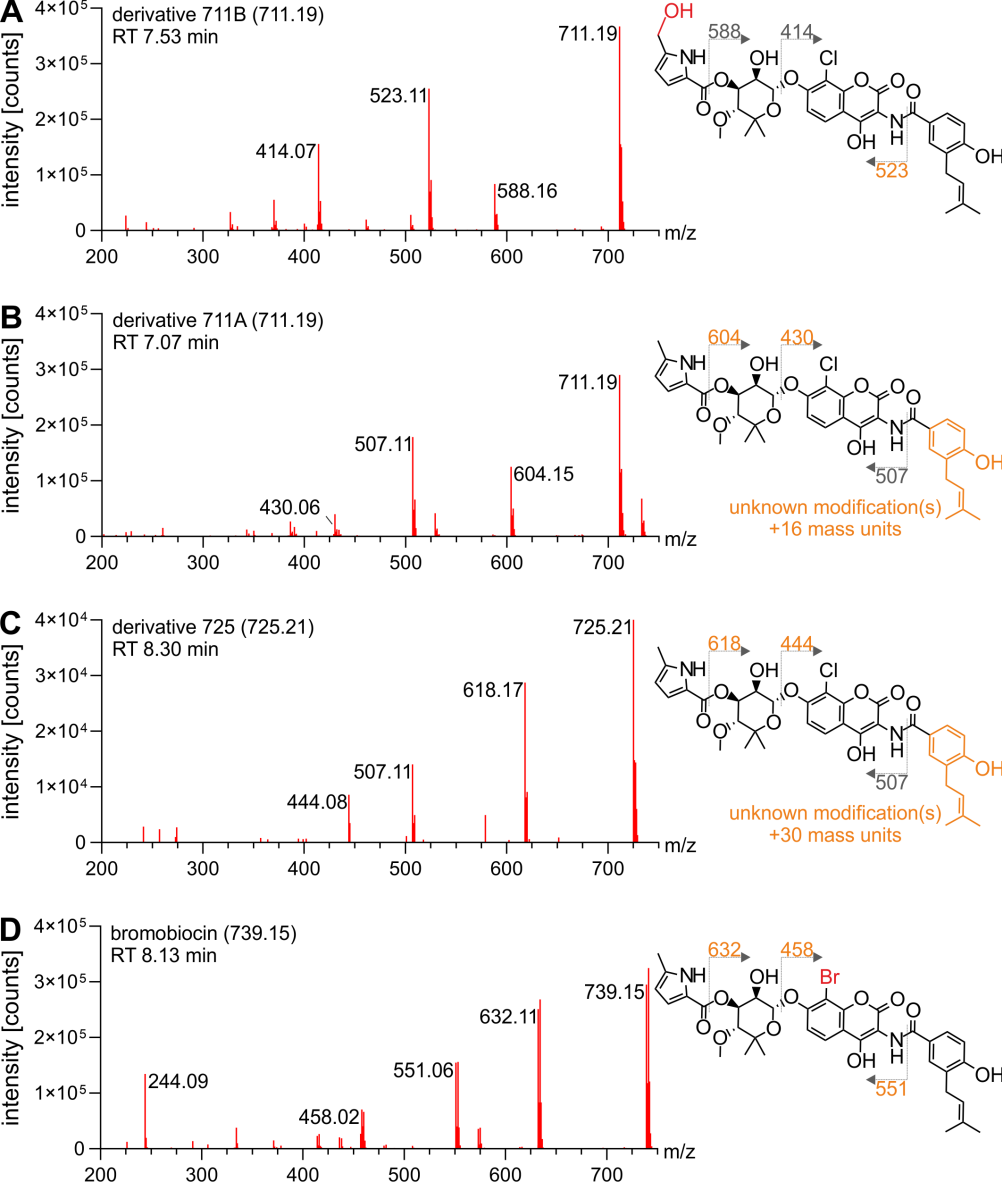
Fragmentation spectra-based localization of modification sites in clorobiocin derivatives. Clorobiocin and clorobiocin-like compounds were extracted from the culture supernatant of *S. roseochromogenes* DS 12.976 and analyzed using LC-MS^E^. The clorobiocin fragmentation spectrum (Fig. 3E) served as a reference to predict fragmentation sites and locations of structural modifications present in identified clorobiocin derivatives. (A–D) The [M-H]^−^ of each parental mass is given in brackets. Fragmentation sites are denoted by dotted lines, and [M-H]^−^ of fragments are indicated in orange when they deviate from the clorobiocin fragmentation spectrum. Structural elements predicted to carry unknown modifications are highlighted in orange. (A) For derivative 711B, localization of the hydroxyl group (shown in red) was determined by NMR analysis. (D) The bromination (shown in red) shows as fragment double peaks of approximately equal abundance with a mass difference of two mass units.

To predict the locations of structural variation, the fragmentation spectra of derivatives were analyzed in detail (Fig. 6), utilizing the annotated clorobiocin fragmentation spectrum as reference (Fig. 3E). In all cases, specific fragment peaks of the derivatives exhibited a mass increase corresponding to the presence of a modification (highlighted in orange), while other fragment peaks remained unaffected. Derivate 711B (Fig. 6A), which was the most abundant derivative, had unaltered 414.07 and 588.16 [M-H]^−^ fragment peaks, while the 507.11 [M-H]^−^ peak was shifted to 523.11 [M-H]^−^, indicating a modification on the 5-methyl-pyrrole 2-carboxyl moiety. After scaling up production and purification, ^1^H NMR analysis revealed that derivative 711B carries an additional hydroxyl group attached to the C-5′ methyl group of the pyrrole moiety. NMR spectra and structure annotation can be found in Fig. S1 and S2 and Table S2.

In case of the 711A derivative (Fig. 6B), the 414.07 and 588.16 [M-H]^−^ fragments incorporated the 16-Da modification, while the 507.11 [M-H]^−^ fragment remained unchanged indicating a modification of ring A. The same pattern was observed for derivative 725, indicating that its 30-Da modification must be located on ring A as well (Fig. 6C). The molecular subnetwork correctly reflected this higher similarity of derivatives 711A and 725 compared to 711B, which is modified on ring C.

### Identification of a novel brominated clorobiocin variant

Compound 739.2 (Fig. 2) drew our attention due to its distinctive isotopic pattern. In both its mass and fragmentation spectrum, it exhibited an isotopic pattern characteristic of a bromination in place of the chlorination (Fig. 6D). In nature, the two isotopes of bromine, ^79^Br and ^81^Br, are approximately equally abundant. Therefore, in mass spectrometry, brominated molecules are characterized by molecular ion peaks of approximately equal intensity that differ by two atomic mass units. This was observed for compound 739.14 [M-H]^−^ (Fig. 6D). The fragmentation spectrum resembled that of clorobiocin (Fig. 3E), albeit with mass shifts indicative of the bromination. We termed this naturally produced derivative, halogenated with bromine instead of chlorine at its C-8′ position of the aminocoumarin moiety, bromobiocin.

### The effect of KBr supplementation on bromobiocin and clorobiocin production

**Fig 7:**
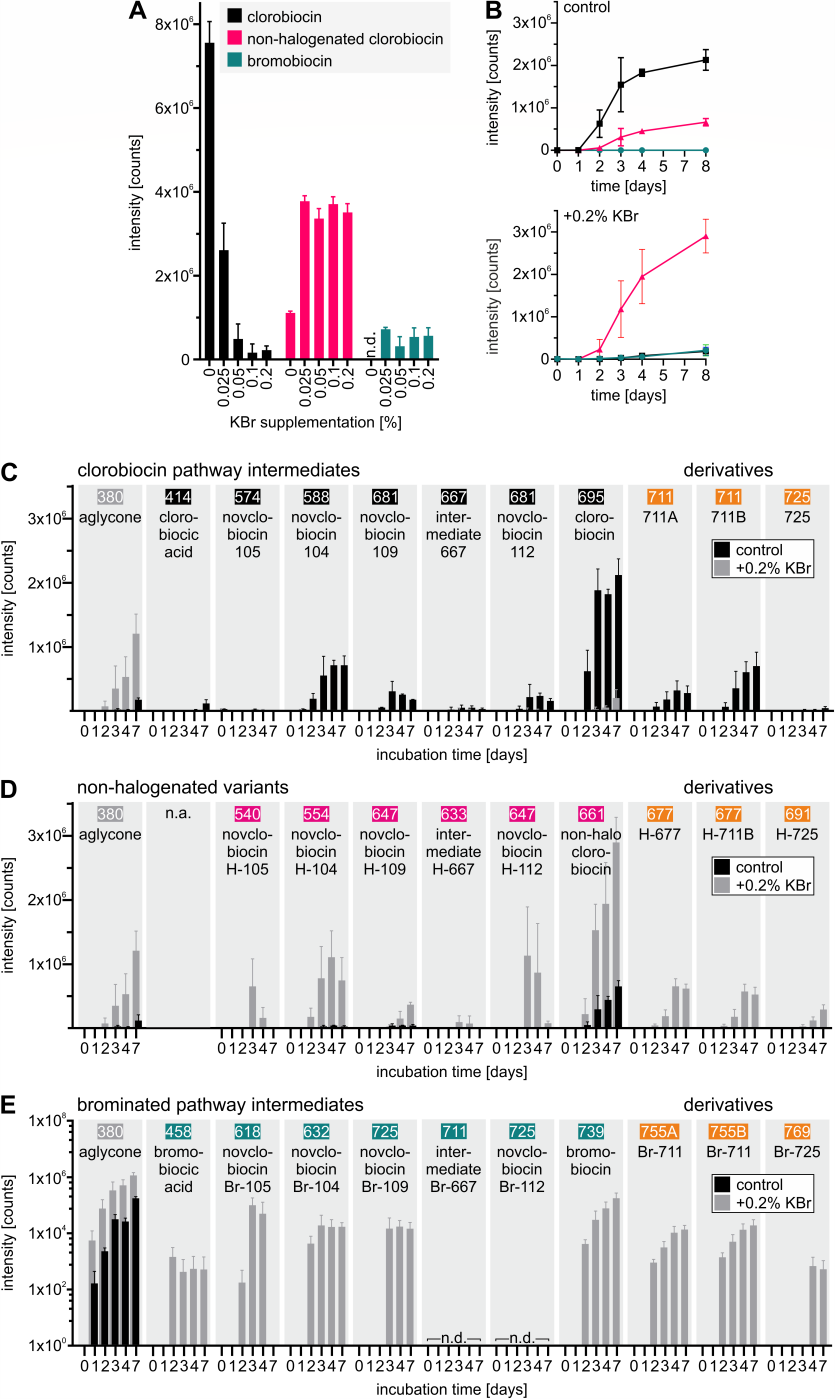
The effect of KBr supplementation on the production of clorobiocin and bromobiocin was investigated. (**A**) *S.roseochromogenes* DS 12.976 was cultured in DSMZ 65 medium (10 mL culture volume) with varying concentrations of KBr. After 7 days of cultivation, the culture supernatants were extracted, and the intensities of clorobiocin, bromobiocin, and non-halogenated clorobiocin were determined by LC-MS^E^ analysis. (**B–E**) Starting from 1 L cultures with or without 0.2% KBr (w/v) supplementation, time course experiments over a 7-day period were performed. (**B**) Intensities of clorobiocin, bromobiocin, and non-halogenated clorobiocin. (**C**) Intensities of clorobiocin, clorobiocin-related compounds, and derivatives. (**D**) Intensities of non-halogenated clorobiocin, non-halogenated related compounds, and derivatives were monitored. (**E**) Intensities of bromobiocin, brominated related compounds, and derivatives. Numbers indicate the [M-H]^−^ of each molecule. Note the different *y*-axes. Data reflect means of an *N* = 3 and standard deviations. n.a., not applicable; n.d., not detected.

Bromobiocin was initially detected at a very low intensity, so we tried to enhance its production through supplementation of the cultivation medium with KBr at various concentrations (Fig. 7A).

While almost no bromobiocin was detected under control conditions, intensities increased when 0.025% KBr (w/v) were added to the cultivation medium. However, higher KBr concentrations did not result in a further increase in bromobiocin intensities and, compared to clorobiocin intensities under control conditions, the detected bromobiocin intensities remained low. Notably, clorobiocin intensities decreased significantly with increasing KBr concentrations. At 0.025% KBr (w/v), clorobiocin intensities were already more than halved, dropping to less than 10% at higher concentrations. Instead, non-halogenated clorobiocin became the predominant metabolite in the culture extracts. Like bromobiocin, its abundance peaked at 0.025% KBr (w/v).

When monitoring the production of clorobiocin, bromobiocin, and non-halogenated clorobiocin over a 7-day period (Fig. 7B), in the absence of KBr, the intensity of non-halogenated clorobiocin increased in parallel to that of clorobiocin reaching approximately one-third its intensity. In the culture supplemented with 0.2% KBr (w/v), clorobiocin intensities stayed low, but non-halogenated clorobiocin was detected at intensities similar to those achieved for clorobiocin under control conditions, suggesting that KBr negatively impacts on the halogenation reaction. A closer look at the intensities of the clorobiocin pathway products (Fig. 7C) and clorobiocin derivatives (Fig. 7D) in cultures with and without 0.2% KBr (w/v) supplementation showed that under control conditions, both clorobiocin and the 711A + B derivatives started to accumulate after approximately 2 days of cultivation. The most abundant pathway intermediates were novclobiocin 104 and novclobiocin 109, which also started to accumulate at that time. In these conditions, the aglycone, clorobiocic acid, and novclobiocin 105 did either not accumulate at all or showed low intensities even after 7 days of incubation. In medium supplemented with KBr, in which clorobiocin production plummeted, clorobiocin pathway products and derivatives were hardly detectable. The clorobiocin aglycone, on the other hand, started to accumulate after 2 days of incubation, and, like non-halogenated clorobiocin, the other non-halogenated versions of the described clorobiocin pathway products (see Fig. S3 for structure annotation) were found with intensities similar to their chlorinated counterparts under control conditions (Fig. 7D). Non-halogenated counterparts of the newly described clorobiocin derivatives 711A, 771B, and 725 were also identified with comparable intensities (see Fig. S4 for structure annotation). Interestingly, the molecule with the mass predicted for the non-halogenated variant of derivative 711B turned out to be modified on the aminocoumarin moiety instead of the 5-methyl-pyrrole 2-carboxyl moiety (Fig. S4). Therefore, it was named derivative H-677 instead. It was also part of the initial clorobiocin spectral family (Fig. 2) that was generated using data from control conditions without KBr supplementation.

Further investigation identified the most brominated pathway intermediates which most likely led to the production of bromobiocin (see Fig. S5 for mass or fragment spectra). As expected, these were only detected in KBr-supplemented cultures and at very low intensities (Fig. 7E). As a result, not all fragmentation spectra could be obtained. Similarly, the newly described clorobiocin derivatives 711A, 711B, and 725 that were found as non-halogenated versions (Fig. S4) were also identified in brominated forms ([M-H]^−^ of 755.14 and 769.16), albeit at very low intensities (Fig. S6).

Finally, to test if other halogens could also be incorporated by the clorobiocin halogenase, *S. roseochromogenes* DS 12.976 cultures were supplemented with 0.1% NaF or KI (w/v). No clorobiocin variants halogenated with iodine or fluorine were detected, and clorobiocin biosynthesis was not affected.

### Antibacterial activity of bromobiocin and clorobiocin derivatives

To further characterize bromobiocin and clorobiocin derivative 711B, their antibacterial activities were investigated. MICs against *E. coli* EP1581 and *B. subtilis* 168 were determined, and disk diffusion assays were performed (Table 1). As we did not succeed in separating bromobiocin from clorobiocin, the antibacterial activity of a 4:3 clorobiocin:bromobiocin mixture was tested (Fig. S7). Clorobiocin, non-halogenated clorobiocin, and novclobiocin 104 were used as reference compounds. Overall, the gram-negative drug efflux-impaired *E. coli* EP1581 strain was more susceptible to clorobiocin and its analogs than the gram-positive *B. subtilis* 168. Interestingly, the clorobiocin/bromobiocin mixture exhibited an antibacterial activity comparable to that of clorobiocin alone. The MICs of both against *E. coli* and *B. subtilis* were 2 and 10 µg/mL*,* respectively, and the inhibition zones in the disk diffusion assay were identical for clorobiocin and the mixture (Fig. S8).

**TABLE 1:** Antibacterial activity of clorobiocin and clorobiocin derivatives

	*E. coli* EP1581		*B. subtilis* 168	
Compound	Inhibition zone (mm)	MIC (µg/mL)	Inhibition zone (mm)	MIC (µg/mL)
Clorobiocin	14	2.0	12	10
Mixture^*b*^ Clorobiocin/bromobiocin	14	2.0	12	10
Derivative 711B	14	5–10	11	14
Derivative H-677	11	>10^*a*^	None	>16^*a*^
Non-halogenated clorobiocin 661	13	6	8	>14^*a*^
Novclobiocin 104	None	>14^*a*^	None	>14^*a*^

^*a*^ Indicates the highest tested concentration for which no MIC was detected.

^*b*^ The 4:3 mixture of clorobiocin and bromobiocin based on MS signal intensity.

However, the 711B derivative had a reduced antibacterial activity compared to clorobiocin, with MIC values between 5 and 10 µg/mL against *E. coli* and 14 µg/mL against *B. subtilis*. The observed inhibition zones for *B. subtilis* were slightly smaller in diameter. Non-halogenated clorobiocin (661) was less active against both strains than clorobiocin. Compared to non-halogenated clorobiocin, the non-halogenated derivative H-677 displayed reduced antibacterial activity against *E. coli* and no activity against *B. subtilis*. Notably, the clorobiocin precursor novclobiocin 104 showed no activity at all, emphasizing the importance of the 5-methyl-pyrrole 2-carboxyl moiety for the antibacterial properties of clorobiocin.

## DISCUSSION

The GNPS web-based mass spectrometry ecosystem allows for the generation of molecular networks that visualize complex metabolomic data sets by clustering structurally related metabolites based on fragmentation spectra similarity. Utilizing this technique, we set out to reinvestigate the biosynthesis of clorobiocin in its natural producer *S. roseochromogenes* DS 12.976 and specifically screen for the presence of natural clorobiocin derivates. Due to their potent antibacterial activity, biomolecular accessibility, and structural similarity, clorobiocin and the related aminocoumarins are well characterized, as are their biosynthetic pathways. However, there are still some open questions regarding clorobiocin biosynthesis, for instance, the precise mechanism of the formation of the 5-methyl-pyrrole 2-carboxyl moiety. By employing an LC-MS^E^-based approach combined with the GNPS platform and fragmentation spectrum analysis, we gained new insights into the clorobiocin biosynthetic pathway and discovered novel natural clorobiocin derivatives. A previous MS-based characterization by Kammerer *et al*. (21) predating publication of the GNPS platform served as a basis for comparison.

### Novel insights into clorobiocin biosynthesis

Through the generation of a molecular network, we visualized and explored the chemical space around clorobiocin present in extracts of culture supernatants of its natural producer. This approach facilitated the identification and quantification of all known intermediates as well as some previously unknown pathway products that follow the formation of the clorobiocin aglycone, providing new insights into clorobiocin biosynthesis.

#### Methylation and acylation of the deoxy sugar noviose during clorobiocin biosynthesis

The final steps in the biosynthesis of the classic aminocoumarins (novobiocin, clorobiocin, and coumermycin A1) entail the methylation of the 4′-OH and the acylation of the 3′-OH of the deoxy sugar moiety. Generally, it is presumed that 4′-OH methylation precedes 3′-OH acylation. Our data reveal that acylation of the clorobiocin precursor can occur without prior CloP-mediated 4′-OH methylation, as evidenced by the detection of compound 667 and novclobiocin 112 in the fermentation broth of *S. roseochromogenes* DS 12.976. To the best of our knowledge, these compounds have not been described as natural constituents of the clorobiocin biosynthetic pathway before. Both are acylated but lack 4′-OH methylation of the deoxy sugar. It is unclear whether these compounds can be further processed by the CloP methyltransferase to produce clorobiocin (via methylation of compound 667) or novclobiocin 109 (via methylation of compound 667), but likely they are dead-end shunt products. The crystal structure and *in silico* docking studies of the homologous NovP methyltransferase, responsible for 4′-OH methylation of the novobiocin deoxy sugar moiety, suggested that NovP is unlikely to tolerate significant modifications of the deoxy sugar, as this moiety was shown to occupy a relatively constricted inner section of the enzyme (26). We therefore hypothesize that CloP is unable to use compound 667 and novclobiocin 112 as substrates.

#### Methylation and transfer of the pyrrole 2-carboxyl moiety of clorobiocin

Two compounds lacking methylation of their pyrrole 2-carboxyl moiety were detected in the culture supernatant of *S. roseochromogenes* DS 12.976, namely 667 and novclobiocin 109. This pyrrole 2-carboxyl moiety originates from _L_-proline, which is activated by CloN4, bound to the carrier protein CloN5 and subsequently oxidized by CloN3. CloN2 mediates the subsequent transfer to CloN1, a second carrier protein (27, 28). For the final assembly of the clorobiocin molecule, it was proposed by Anderle *et al*. that transfer and methylation of the pyrrole 2-carboxyl moiety occur simultaneously, necessitating the coordinated action of CloN1, the methyltransferase CloN6, and the acyltransferase CloN7 (29). Considering the presence of compound 667 and novclobiocin 109 in the culture supernatant of *S. roseochromogenes*, it appears that methylation and transfer of the pyrrole 2-carboxyl moiety can occur in two separate enzymatic steps, as proposed originally by Heide. (19). Since it has been demonstrated that CloN6 most likely is not able to facilitate methylation of the pyrrole 2-carboxyl moiety once it is transferred to the deoxy sugar (29), our data imply that the transfer of the pyrrole 2-carboxyl moiety does not necessarily involve methylation, resulting in the production of clorobiocin as well as some novclobiocin 109. An accumulation of novclobiocin 109 was previously reported for a *cloN6*^−^ mutant, demonstrating that, in principle, CloN7 can facilitate the transfer of the pyrrole 2-carboxyl moiety independently from CloN6 (30). Since novclobiocin 109 has significantly lower antibacterial activity (30), the coupling of CloN6-mediated methylation to CloN7-mediated transfer appears beneficial since it would prevent a premature export, thus ensuring a higher clorobiocin production.

#### Biosynthesis of non-halogenated clorobiocin

We found that a significant portion of the metabolic output of the clorobiocin biosynthetic gene cluster is non-halogenated clorobiocin which accounted for 15%–30% of the total clorobiocin intensity depending on cultivation conditions. Kammerer *et al*. also detected non-halogenated clorobiocin in culture supernatants of *S. roseochromogenes* DS 12.976 (21) and postulated that it is the last pathway intermediate preceding clorobiocin. Later, it was generally assumed that Clo-hal exclusively halogenates the clorobiocin aglycone (12, 31, 32). If this is the case, non-halogenated clorobiocin could be an alternative end product of the clorobiocin biosynthetic pathway that results from the substrate promiscuity of CloM, CloP, CloN7, and CloN6 that allows them to process non-chlorinated substrates (13).

The clorobiocin molecule is most likely evolved from a non-halogenated developmental predecessor. Similarly, natural novobiocin variants lacking methylation of their aminocoumarin moieties were found in *Actinobacteria* and are considered developmental predecessors of novobiocin (33). Many of the enzymes within the aminocoumarin biosynthetic gene clusters likely evolved processing unmodified aminocoumarin moieties and retained affinity for the respective substrates even when halogenated (clorobiocin) or methylated (novobiocin) aminocoumarin variants emerged. As a result, CloM and other downstream enzymes continue to facilitate the production of non-halogenated clorobiocin despite its inferior anti-gyrase activity (13). Accumulation of non-halogenated clorobiocin was previously reported for a *clo-hal*-deficient strain (13), yet, to our knowledge, it has not been discussed as a natural end product of the clorobiocin pathway.

### Novel clorobiocin derivatives

#### Discovery of clorobiocin derivatives 711A, 711B, and 725

In their MS-based investigation of the clorobiocin biosynthetic pathway, Kammerer *et al*. reported a novel clorobiocin derivative and postulated, based on fragmentation spectra analysis, a potential hydroxylation on the 5-methyl-pyrrole 2-carboxyl moiety (21). We successfully re-identified this natural derivative in the clorobiocin spectral family (711B) and conclusively confirmed its structure through 1H NMR analysis (Fig. S1). Activity assays verified that derivative 711B retained antibacterial activity, albeit slightly lower than clorobiocin. Furthermore, Kammerer *et al*. (21) speculated that there might be further clorobiocin derivatives with a second methyl ([M-H]^−^ = 709) or 5-methyl-pyrrole 2-carboxyl ([M-H]^−^ = 800) moiety on their deoxy sugar moieties, but the respective molecules were not detected. We did not detect these modifications either. However, we identified two other previously unpredicted clorobiocin derivates (711A and 725). Both exhibited fragmentation spectra indicating structural modifications on ring A. Unfortunately, substance yields were too low for structure elucidation or activity assays. However, it may be worthwhile in future studies to investigate those derivatives further since some genetically engineered ring A modifications, such as an additional double bond in novclobiocin 225, have been reported to increase the anti-gyrase activity of clorobiocin (34).

#### Discovery of bromobiocin and the effect of KBr supplementation

Within the clorobiocin spectral family, we identified compound 739 as a natural clorobiocin derivative halogenated with bromine instead of chlorine and named it “bromobiocin.” KBr supplementation activated bromobiocin production but led to a disproportional reduction in clorobiocin levels, with non-halogenated clorobiocin being the most prevalent metabolite. Consequently, non-chlorinated and brominated clorobiocin precursors and derivatives were detected.

Clo-hal halogenates the clorobiocin aglycone and is a flavin-dependent halogenase. This enzyme family is involved in the halogenation of numerous natural products including vancomycin, pyoluteorin, and rebeccamycin (35). Except for fluoride, flavin-dependent halogenases, in general, are known to introduce any common halides (Cl, Br, and I) (36–38), and several of the enzymes have been shown to accept more than one halide as substrate when cultivation media are supplemented accordingly. In such cases, the halide preference is specific to the enzyme. For instance, in the vancomycin-type glycopeptide balhimycin, bromine and chlorine (39) are incorporated equally frequently, whereas the halogenation reaction in rebeccamycin (40) and pyrrolnitrin (41) was shown to favor chlorine over bromine. Furthermore, Dorrstein *et al*. observed that the incorporation of a sterically larger bromine into pyoluteorin is associated with a significantly reduced turnover rate compared to the incorporation of chlorine (42). Similarly, we found that Clo-hal is capable of using both bromide and chloride. Considering the difference in chloride and bromide concentrations in the medium, our data suggest that Clo-hal has a higher affinity for bromide, explaining the drastic decrease in clorobiocin when comparably small concentrations of KBr are added to the cultivation medium. However, the lower bromobiocin production also suggests that the overall turnover rate of Clo-hal is greatly reduced when utilizing bromide. This is also supported by the observed accumulation of the precursor clorobiocin aglycone when KBr is supplemented. With KBr supplementation, an accumulation of non-halogenated clorobiocin is observed, which can be explained by the previously described ability of CloM and the other downstream enzymes to process non-halogenated substrates. Given that there is little accumulation of pathway intermediates under KBr supplementation conditions, the “non-halogenated” pathway seems to be more efficient than the “brominated.” For some natural products, the incorporation of different halogens has been observed to alter their activity. For instance, bromobalhimycin was reported to be twice as effective against *Enterococcus faecium* compared to its chlorinated counterpart, while it was significantly less effective against *Staphylococcus aureus* (39). The activity of bromobiocin against *B. subtilis* and efflux-impaired *E. coli* was found to be comparable to that of clorobiocin. However, it may be beneficial to test bromobiocin against a broader range of bacteria, particularly pathogens, to identify potential species-dependent effects.

### Conclusion

In summary, our immediate metabolite-driven workflow proves highly effective for investigating biosynthetic pathways and uncovering new natural products. Studying clorobiocin biosynthesis, arguably one of the best-understood metabolic pathways in streptomycetes, by LC-MS^E^ analysis of extracted culture supernatants, we successfully elucidated and expanded the known chemical space of clorobiocin, revealing novel intermediates and derivatives. Based on the detection of natural products that vary in the noviose and pyrrole 2-carboxyl moieties as well as halogenation state, we propose that the known enzymes, due to promiscuity, realize parallel biosynthetic pathways. When adapted for uncharted bioactive compounds, based on the structural elucidation of a few compounds amenable to NMR analysis, this analytic strategy allows for the rapid identification of critical pathway intermediates, shunt products, and derivatives, even if those are only present in limited quantities, providing valuable insights into biosynthetic pathways.

## Data Availability

The metabolomic data presented in this study have been deposited into the Global Natural Products Social Molecular Networking Library as MassIVE dataset MSV000093926.

## SUPPLEMENTAL MATERIAL

Supplemental tables and figures spectrum.00423-24-s0001.pdf Tables S1 and S2; Fig. S1-S8.

ASM does not own the copyrights to Supplemental Material that may be linked to, or accessed through, an article. The authors have granted ASM a non-exclusive, world-wide license to publish the Supplemental Material files. Please contact the corresponding author directly for reuse.
