# Supplementary material for: A metabolomics perspective on clorobiocin biosynthesis: discovery of bromobiocin and novel derivatives through LC-MSE-based molecular networking
Source: Microbiol Spectr. 2024 Jun 12;12(7):e00423-24. doi: 10.1128/spectrum.00423-24 (PMC11218499; doi:10.1128/spectrum.00423-24)
Supplement: Supplemental tables and figures — Tables S1 and S2; Fig. S1-S8. [file spectrum.00423-24-s0001.pdf]

Supplementary File

A metabolomics perspective on clorobiocin biosynthesis: discovery of bromobiocin and novel derivatives through LC-MS^E-based molecular networking

Niklas B. M. Janzing[1], Maurice Niehoff[2], Wolfram Sander[2], Christoph H. R. Senges[1], Sina Schäkermann[1], Julia E. Bandow[1*]

[1]Applied Microbiology, Faculty of Biology and Biotechnology, Ruhr University Bochum, Germany

[2]Organic Chemistry II, Faculty of Chemistry and Biochemistry, Ruhr University Bochum, Germany

*Corresponding author: Ruhr University Bochum, Applied Microbiology, Universitätsstraße 150, 44801 Bochum, Germany. Phone: +49-234-32-23102.

E-Mail: julia.bandow@rub.de

Supplementary information

Supplementary table S1: Identification of compounds within the clorobiocin spectral family.

Supplementary table S2: ^{1}H NMR shifts of clorobiocin derivative 711B.

Supplementary figure S1: ^{1}H NMR spectrum of clorobiocin derivative 711B.

Supplementary figure S2: COSY of clorobiocin derivative 711B.

Supplementary figure S3: Fragmentation spectra of non-halogenated clorobiocin pathway intermediates and shunt products.

Supplementary figure S4: Fragmentation spectra-based determination of modification sites in non-halogenated clorobiocin derivatives.

Supplementary figure S5: Parent mass and fragmentation spectra of bromobiocin pathway intermediates and shunt products.

Supplementary figure S6: Fragmentation spectra-based determination of modification sites in brominated clorobiocin derivatives.

Supplementary figure S7: Integrated total ion chromatograms of clorobiocin and clorobiocin-related compounds.

Supplementary figure S8: Disk diffusion assay of clorobiocin and clorobiocin-related compounds.

Supplementary table S1

Table S1: Identification of compounds within the clorobiocin spectral family (Fig. 2) detected in the culture supernatant of *S. roseochromogenes* DS 12.976. Formulas and calculated monoisotopic masses $[M\text{-}H]^-$ are presented for products and intermediates of the clorobiocin, non-halogenated clorobiocin, and bromobiocin pathways, including for derivative 711B, whose structure was elucidated. The observed masses, mass errors, measured retention times (RT), and measured chemical cross sections (CCS) are listed.

Name	Formula	Calculated monoisotopic mass	Observed mass	Error [ppm]	RT [min]	CCS [Å^2]
Clorobiocic acid	$C_{21}H_{18}ClNO_6$	414.07499	414.07505	-0.14	6.97	192.23
Novclobiocin 105	$C_{28}H_{30}ClNO_{10}$	574.14855	574.14837	0.31	6.58	236.61
Novclobiocin 104	$C_{29}H_{32}ClNO_{10}$	588.16420	588.16430	-0.17	7.26	241.71
Compound 667	$C_{33}H_{33}ClN_2O_{11}$	667.17001	667.17056	-0.82	7.29	259.34
Novclobiocin 112	$C_{34}H_{35}ClN_2O_{11}$	681.18566	681.18630	-0.94	7.43	257.98
Novclobiocin 109	$C_{34}H_{35}ClN_2O_{11}$	681.18566	681.18640	-1.09	7.99	259.08
Clorobiocin	$C_{35}H_{37}ClN_2O_{11}$	695.20131	695.20245	-1.64	8.13	258.50
Novclobiocin H-105	$C_{28}H_{31}NO_{10}$	540.18752	540.18754	-0.04	5.30	234.72
Novclobiocin H-104	$C_{29}H_{33}NO_{10}$	554.20317	554.20282	0.63	7.02	238.83
Compound H-667	$C_{33}H_{34}N_2O_{11}$	633.20898	633.20829	1.09	7.14	258.18
Novclobiocin H-112	$C_{34}H_{36}N_2O_{11}$	647.22463	647.22407	0.87	7.57	238.32
Novclobiocin H-109	$C_{34}H_{36}N_2O_{11}$	647.22463	647.22446	0.26	7.80	256.60
Non-halogenated clorobiocin	$C_{35}H_{38}N_2O_{11}$	661.24028	661.24043	-0.23	7.95	256.66
Bromobiocic acid	$C_{21}H_{18}BrNO_6$	458.02447	458.02267	3.93	7.00	189.51
Novclobiocin Br-105	$C_{28}H_{30}BrNO_{10}$	618.09803	618.09714	1.44	6.88	234.09
Novclobiocin Br-104	$C_{29}H_{32}BrNO_{10}$	632.11368	632.11276	1.46	7.31	240.55
Compound Br-667	$C_{33}H_{33}BrN_2O_{11}$	711.11950	-	-	-	-
Novclobiocin Br-112	$C_{34}H_{35}BrN_2O_{11}$	725.13515	-	-	-	-
Novclobiocin Br-109	$C_{34}H_{35}BrN_2O_{11}$	725.13515	725.13522	-0.10	7.46	257.72
Bromobiocin	$C_{35}H_{37}BrN_2O_{11}$	739.15080	739.15071	0.12	8.13	257.62
Derivative 711A	-	-	711.19648	-	7.07	257.27
Derivative 711B	$C_{35}H_{37}ClN_2O_{12}$	711.19623	711.19697	-1.04	7.54	250.61
Derivative 677B	-	-	677.23478	-	7.34	251.01
Derivative 725	-	-	725.21335	-	8.30	265.71

Supplementary table S2

Table S2: ^{1}H NMR shifts of clorobiocin derivative 711B. The sample was measured in MeOH-d4 (approx. 3.5 µmol/mL) on a 400 MHz NMR spectrometer (Bruker). The assignment of peaks is based on literature [1, 2]

Position	δ_H [ppm] (*J* in Hz)
1	1.22 (3H, s)
2	3.73 (1H, d, *J* = 10.1 Hz)
3	5.76 (1H, dd, *J* = 10.1, 3.1 Hz)
4	4.34 (1H, “t”, *J* = 2.2 Hz)
5	5.68 (1H, “d”, *J* = 1.2 Hz)
6	3.52 (3H, s)
7	1.37 (3H, s)
8	6.96 (1H, d, *J* = 3.7 Hz)
9	6.19 (1H, d, *J* = 3.7 Hz)
10	4.59 (2H, s)
11	7.21 (1H, d, *J* = 8.8 Hz)
12	7.91 (1H, d, *J* = 8.7 Hz)
13	7.77 (1H, s)
14	7.71 (1H, d, *J* = 8.4 Hz)
15	6.80 (1 H, d *J* = 8.3 Hz)
16	3.34 (2H, “d”, *J* = 7.5 Hz)
17	5.35 (1H, t, *J* = 7.3 Hz)
18, 19	1.74 (6H, s)

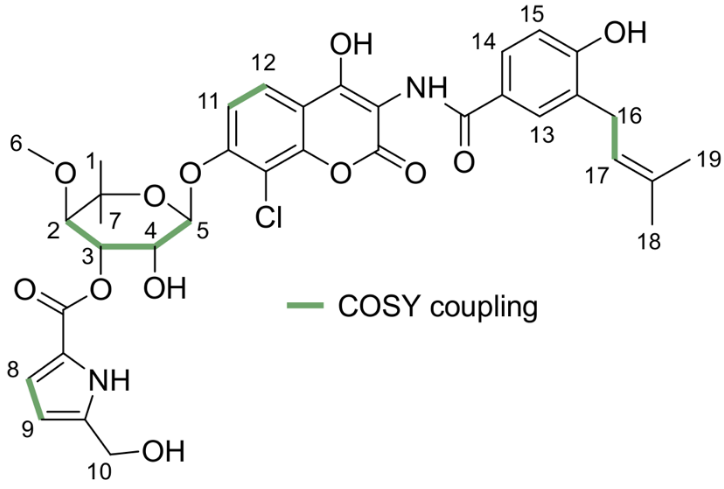

Supplementary figure S1:

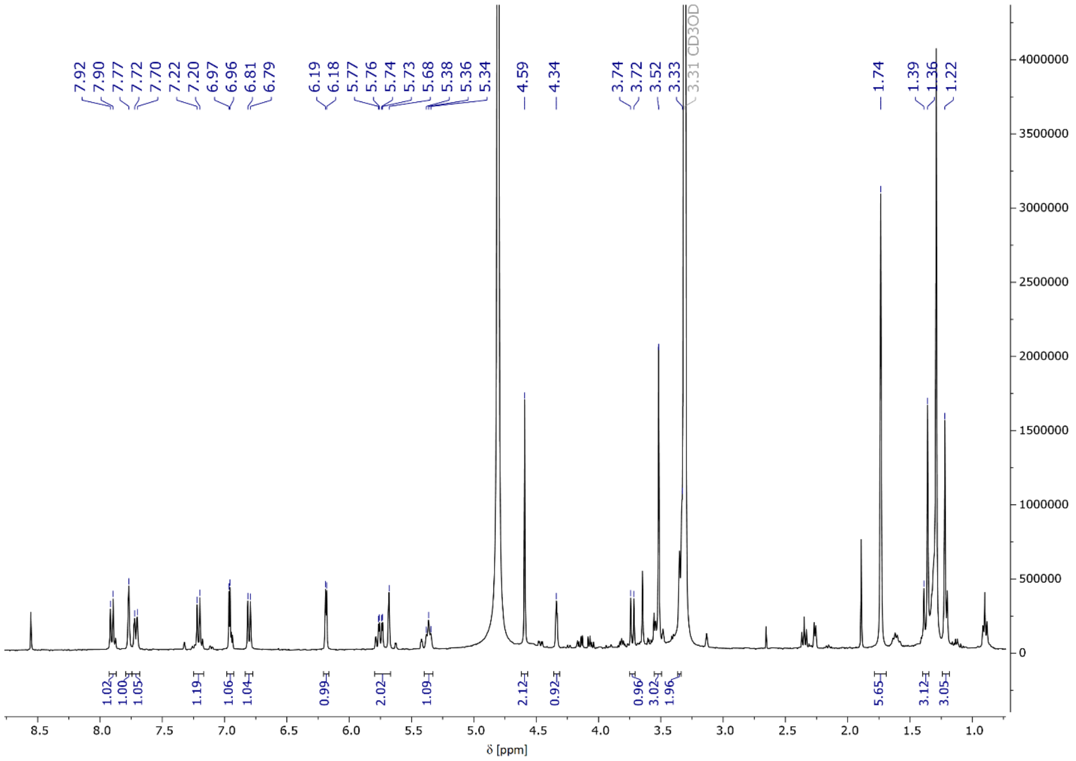

FIG S1 ^{1}H NMR spectrum of clorobiocin derivative 711B in CD_3OD (400 MHz).

Supplementary figure S2:

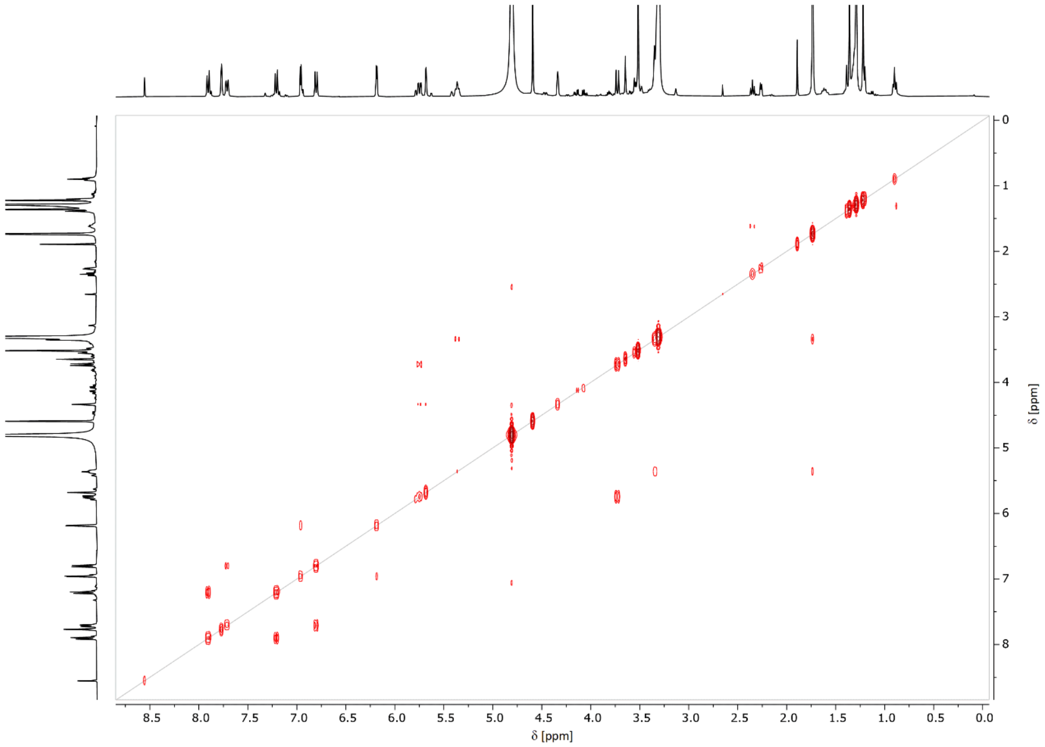

FIG S2 COSY of clorobiocin derivative 711B in CD_3OD (400 MHz).

Supplementary figure S3:

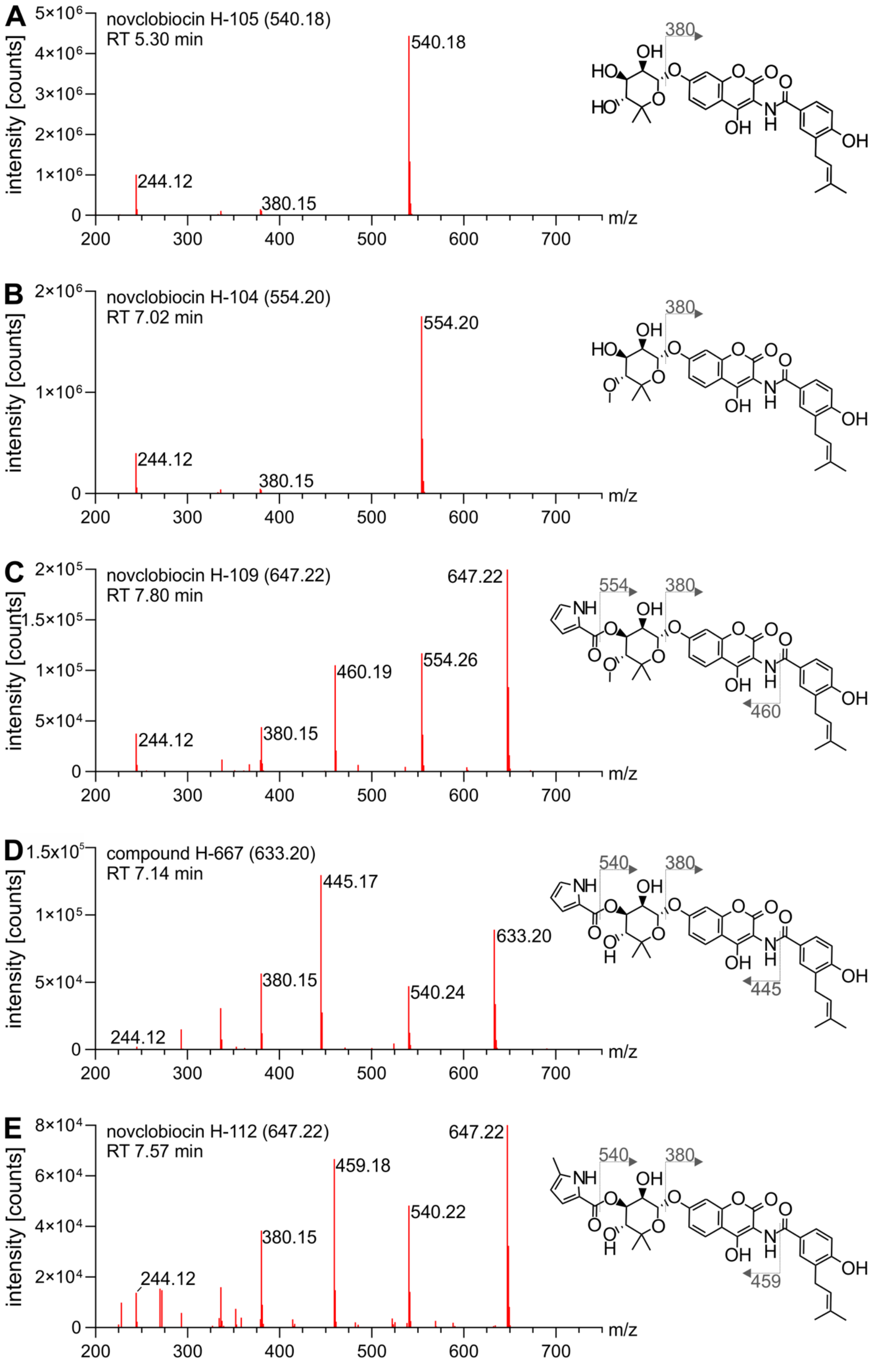

FIG S3 Fragmentation spectra of non-halogenated clorobiocin pathway intermediates and shunt products. *S. roseochromogenes* DS 12.976 was cultivated in DSMZ 65 medium supplemented with 0.2% KBr (w/v) for 7 days. Extracts from the culture supernatant were analyzed using LC-MS^E. Fragment peaks of detected compounds were assigned to proposed structures using the clorobiocin fragmentation spectrum (Fig. 3E) as reference. The $[M\text{-}H]^-$ of each parental mass is given in brackets. Fragmentation sites are denoted by dotted lines and $[M H]^-$ of fragments are indicated.

Supplementary figure S4:

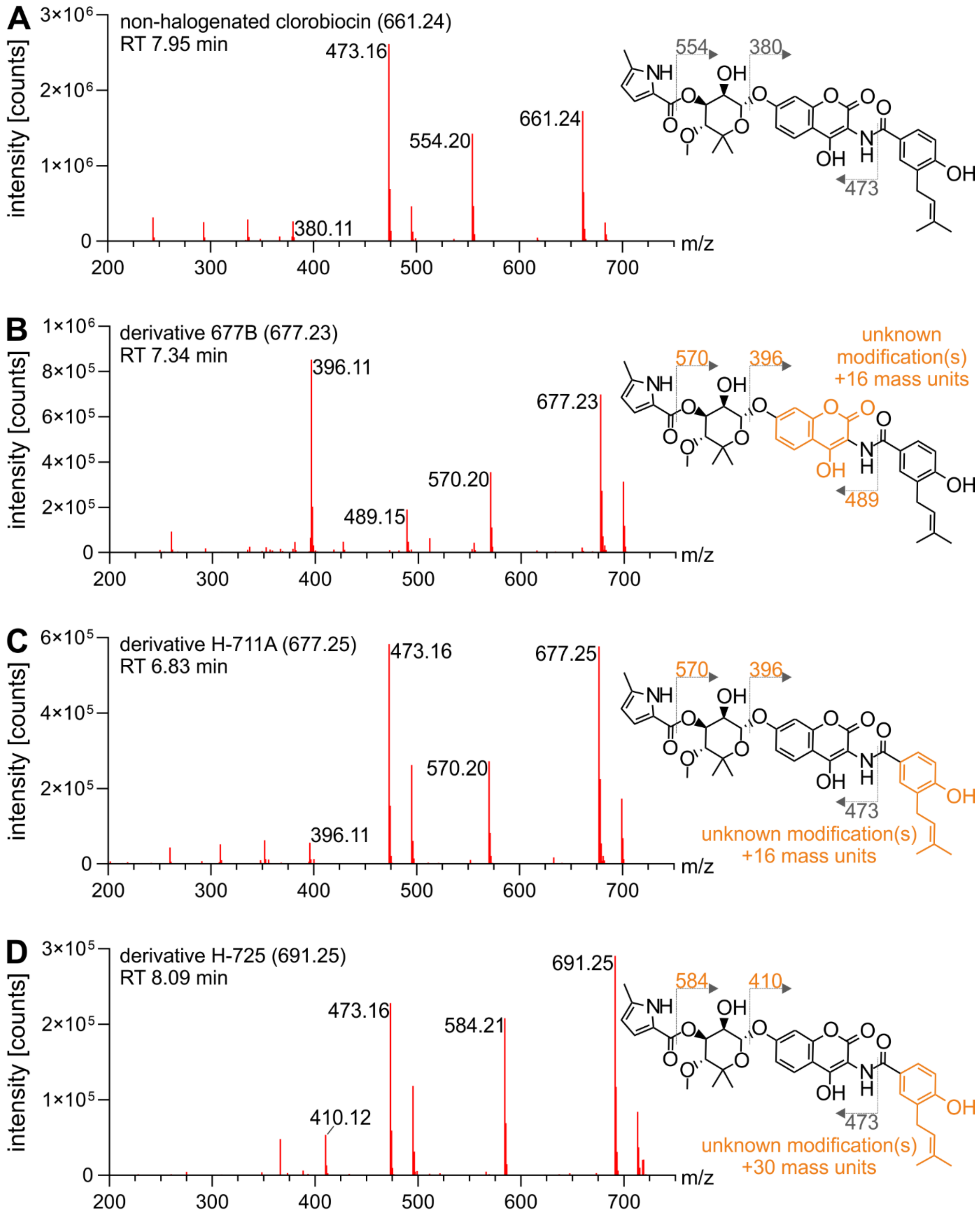

FIG S4 Fragmentation spectra-based determination of modification sites in non-halogenated clorobiocin derivatives. *S. roseochromogenes* DS 12.976 was cultivated in DSMZ 65 medium supplemented with 0.2% KBr (w/v) for 7 days. Extracts from the culture supernatant were analyzed using LC-MS^E. A-D) The fragmentation spectrum of non-halogenated clorobiocin (A) served as a reference to locate structural modifications present in identified derivatives. Fragmentation sites are denoted by dotted lines and $[M-H]^-$ of fragments are indicated in orange if they deviate from the

fragmentation spectrum of non-halogenated clorobiocin. Structural elements that are predicted to carry unknown modifications are highlighted in orange.

Supplementary figure S5:

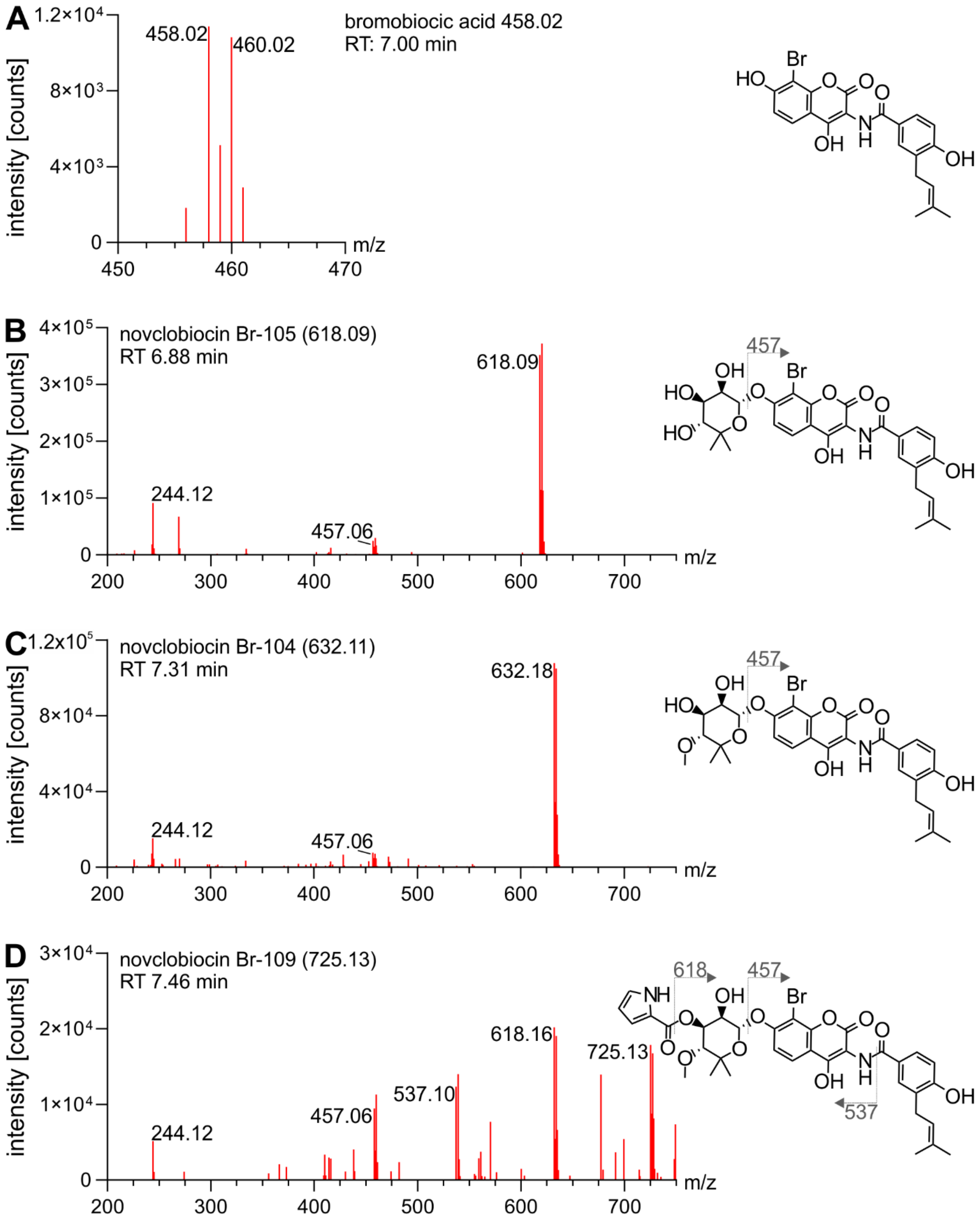

FIG S5 Parent mass and fragmentation spectra of bromobiocin pathway intermediates and shunt products. *S. roseochromogenes* DS 12.976 was cultivated in DSMZ 65 medium supplemented with 0.2% KBr (w/v) for 7 days. Extracts from the culture

supernatant were analyzed using LC-MS^E. The $[M-H]^-$ of each parental mass is given in brackets. Fragmentation sites are denoted by dotted lines and $[M H]^-$ of fragments are indicated.

Supplementary figure S6:

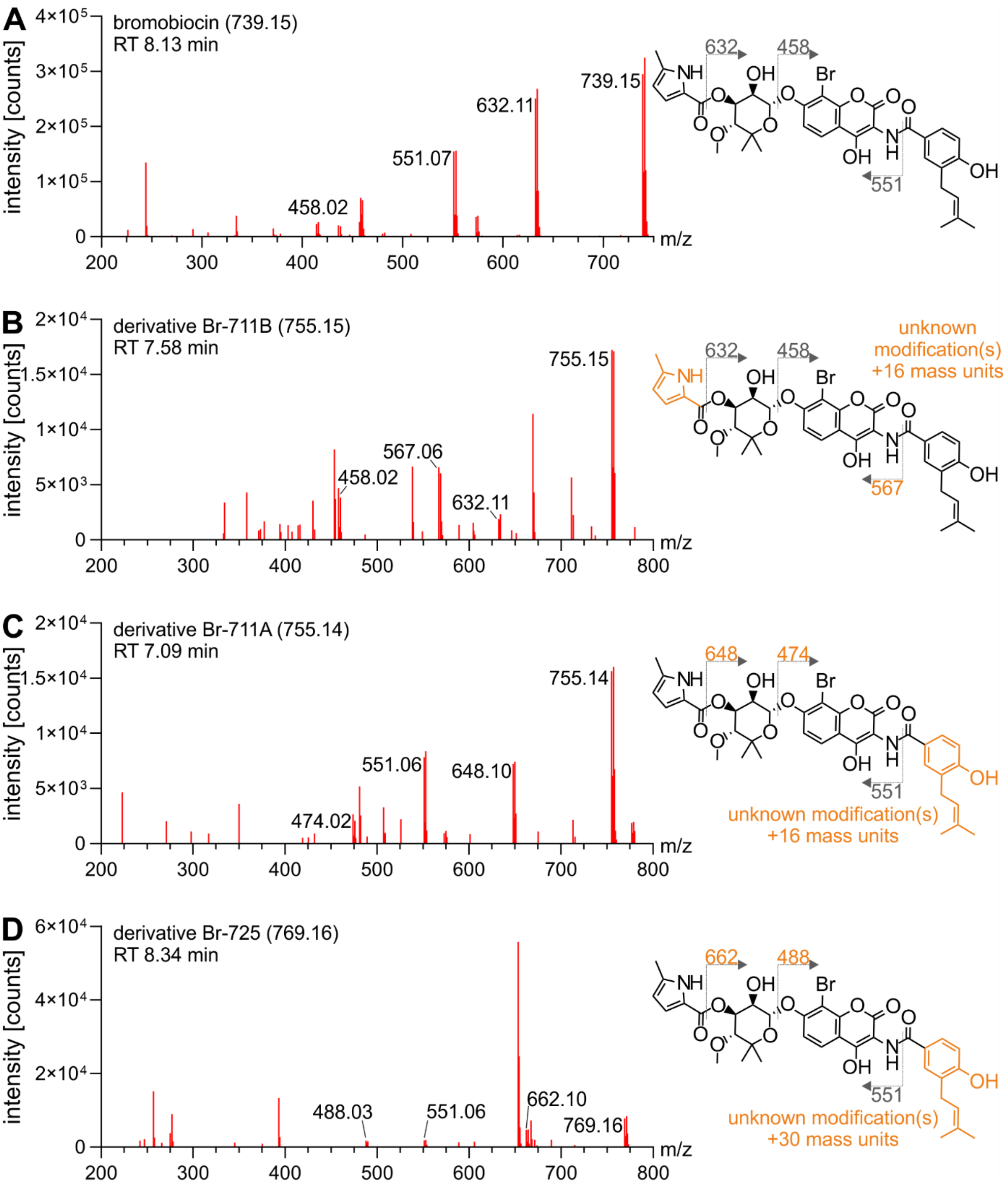

FIG S6 Fragmentation spectra-based determination of modification sites in brominated clorobiocin derivatives. *S. roseochromogenes* DS 12.976 was cultivated in DSMZ 65 medium supplemented with 0.2% (w/v) KBr for 7 days. Extracts from the culture supernatant were analyzed using LC-MS^E. A-D) The fragmentation spectrum of

bromobiocin (A) served as a reference to locate structural modifications present in identified bromobiocin derivatives. Fragmentation sites are denoted by dotted lines and [M-H]$^-$ of fragments are indicated in orange if they deviate from the bromobiocin fragmentation spectrum. Structural elements that are predicted to carry unknown modifications are highlighted in orange.

Supplementary figure S7:

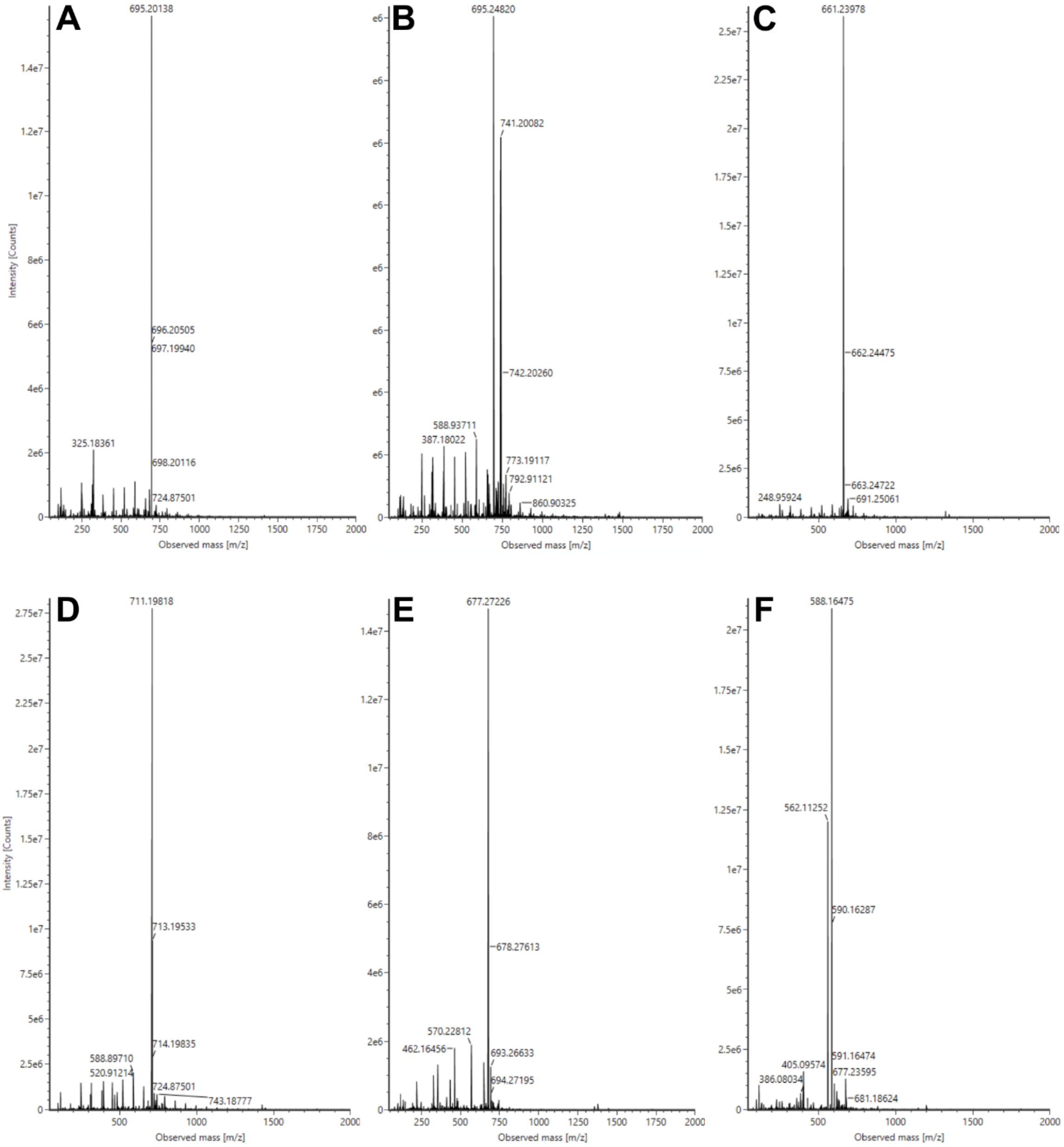

FIG S7 Integrated total ion chromatograms of A) clorobiocin and clorobiocin-related compounds that were isolated from the culture supernatant of *S. roseochromogenes* DS 12.976 and used for antibacterial testing (Table 3): B) clorobiocin/bromobiocin mixture, C) non-halogenated clorobiocin, D) clorobiocin derivative 711B, E) non-halogenated derivate H-677, F) novclobiocin 104.

Supplementary figure S8:

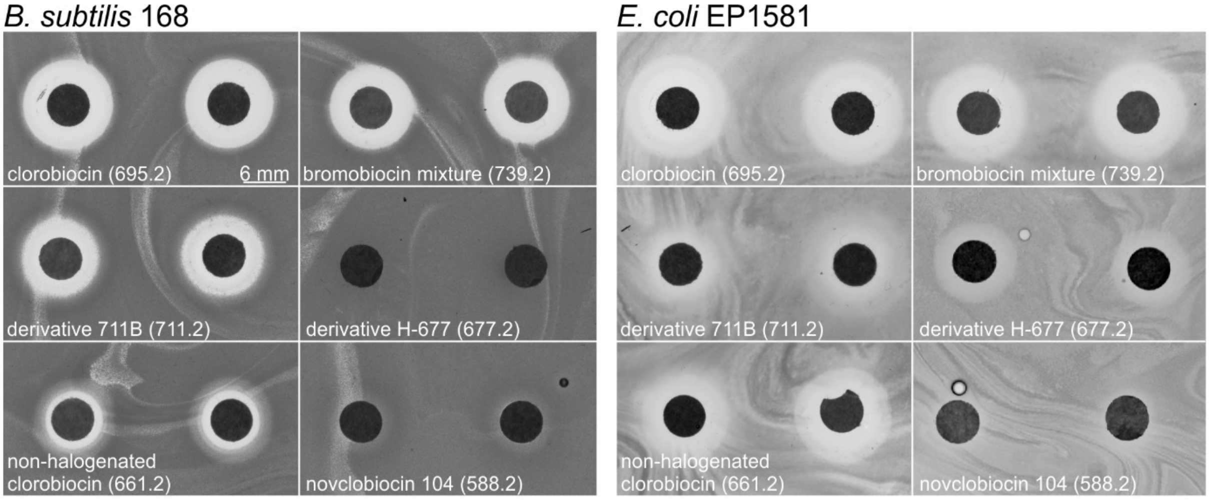

FIG S8 Disk diffusion assay of clorobiocin and clorobiocin-related compounds that were isolated from the culture supernatant of *S. roseochromogenes* DS 12.976. Filter papers with 10 µg of each test substance were placed onto agar plates inoculated with *B. subtilis* 168 or *E. coli* EP1581. The agar plates were incubated overnight at 37°C before inhibition zone diameters were measured. The $[M\text{-}H]^-$ of each molecule is given in brackets.

References

[1] Eustáquio, A.S., Gust, B., Luft, T., Li, S.-M., Chater, K.F., Heide, L., Clorobiocin biosynthesis in *Streptomyces*: identification of the halogenase and generation of structural analogs. *Chem Biol* 2003, 10, 279–288.

[2] Yasui, E., Tsuda, J., Ohnuki, S., Nagumo, S., Selective mono-reduction of pyrrole-2,5 and 2,4-dicarboxylates. *Chem Pharm Bull (Tokyo)* 2016, 64, 1262–1267.
